# Design, Synthesis, and Antibacterial Evaluation of Novel Ocotillol Derivatives and Their Synergistic Effects with Conventional Antibiotics

**DOI:** 10.3390/molecules26195969

**Published:** 2021-10-01

**Authors:** Doudou Zhang, Yucheng Cao, Kaiyi Wang, Zhuoyue Shi, Ruodong Wang, Qingguo Meng, Yi Bi

**Affiliations:** School of Pharmacy, Key Laboratory of Molecular Pharmacology and Drug Evaluation, Ministry of Education, Collaborative Innovation Center of Advanced Drug Delivery System and Biotech Drugs in Universities of Shandong, Yantai University, Yantai 264005, China; koikoi1998@yeah.net (D.Z.); cchm1187829@163.com (Y.C.); wangky_1994@163.com (K.W.); shizhuoyue2021@163.com (Z.S.); wangruodong2021@163.com (R.W.)

**Keywords:** ocotillol-type derivatives, HA-MRSA, antibiotics, structural modification, synergistic effects

## Abstract

The improper use of antibiotics has led to the development of bacterial resistance, resulting in fewer antibiotics for many bacterial infections. Especially, the drug resistance of hospital-acquired methicillin-resistant *Staphylococcus aureus* (HA-MRSA) is distinctly serious. This research designed and synthesized two series of 3-substituted ocotillol derivatives in order to improve their anti-HA-MRSA potency and synergistic antibacterial activity. Among the synthesized compounds, **20**–**31** showed minimum inhibitory concentration (MIC) values of 1–64 µg/mL in vitro against HA-MRSA 18–19, 18–20, and *S. aureus* ATCC29213. Compound **21** showed the best antibacterial activity, with an MIC of 1 μg/mL and had synergistic inhibitory effects. The fractional inhibitory concentration index (FICI) value was 0.375, when combined with chloramphenicol (CHL) or kanamycin (KAN). The structure–activity relationships (SARs) of ocotillol-type derivatives were also summarized. Compound **21** has the potential to be developed as a novel antibacterial agent or potentiator against HA-MRSA.

## 1. Introduction

Globally, most bacteria (e.g., *Mycobacterium tuberculosis*, *Escherichia coli*, and *Staphylococcus aureus*) can cause severe human diseases (e.g., tuberculosis, sepsis, and skin infections) [1,2]. Because of the improper use and abuse of antibiotics, many bacteria have become resistant or even cross-resistant to many antibacterial drugs on the market, such as methicillin, chloramphenicol (CHL), and kanamycin (KAN) [3,4]. Diseases and deaths caused by drug-resistant bacterial infections have resulted in great losses to human health and property [5]. The World Health Organization prioritized 20 drug-resistant bacteria on the basis of indicators, such as mortality, resistance rates, health care burden, 10-year resistance trends, and transmissibility, which showed that methicillin-resistant *Staphylococcus aureus* (MRSA) and vancomycin-resistant *Enterococcus* (VRE) ranked the highest [6].

**Figure 1:**
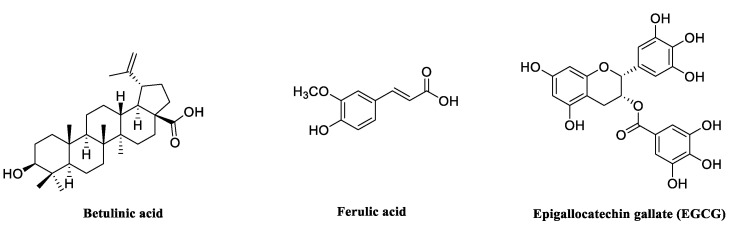
Structures of betulinic acid, ferulic acid, and epipallocatechin gallate (EGCG).

Natural products are important in the development of new drugs. Over the past four decades, medicinal chemists have become increasingly interested in the development of new drugs derived from natural products [7,8]. One of the strategies to treat diseases caused by drug-resistant bacterial infections is to combine existing antibiotics with phytochemicals. Most phytochemicals possess antimicrobial activity and when these plant-derived compounds are used in combination with conventional antibiotics, they can achieve better antibacterial effects and prevent the development of bacterial resistance [9,10,11]. Betulinic acid isolated from the leaves of *Vitex negundo* reduces the MIC of methicillin against MRSA to 1/64 × MIC. When ferulic acid from *Phenylpropanoids* was combined with amikacin, a synergistic antibacterial effect was noticed against *Staphylococcus aureus* NCIM 5021 with a fractional inhibitory concentration index (FICI) of 0.16. Epipallocatechin gallate (EGCG), a principal ingredient of tea, drives the anti-MRSA MIC of imipenem from 128 μg/mL to 8 μg/mL. Therefore, it is necessary to develop alternative natural or combination drug therapies [12,13,14,15] (Figure 1).

**Figure 2:**
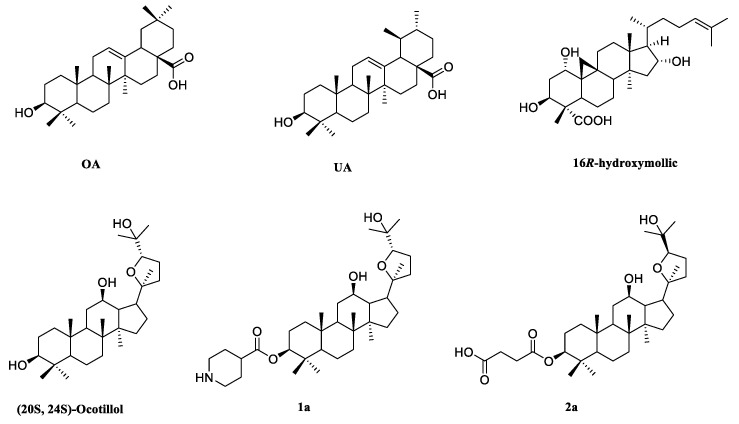
Structures of OA, UA, 16*R*-hydroxymollic, (20*S*, 24*S*)-ocotillol, compounds **1a** and **2a**.

Natural active ingredients derived from terpenoids have good antibacterial activity against MRSA and VRE, such as oleanolic acid (OA) from *Salvia officinalis*, ursolic acid (UA) from *Alstonia scholaris*, and 16R-hydroxymollic from *Acalypha communis* [16,17] (Figure 2). Ginseng is a perennial herb, mainly distributed in China, Russia, and Korea [18]. Ginsenosides and their metabolites (such as G-Rb1, G-Rb2, G-Rd, G-Re, and G-Rg1) have good effects on the human nervous system, cardiovascular system, and endocrine system, among others [18,19,20]. Ocotillol-type ginsenosides found in *Panax quinquefolius* L., *Vietnamese* ginseng, and *Panax japonicus* have a wide range of excellent pharmacological activities (including anti-ischemic, anti-inflammatory, and antitumor activities) [21]. Our preliminary research found that ocotillol-type ginsenosides have good antibacterial activity, such as (20*S*, 24*S*)-ocotillol with an MIC value of 8 µg/mL against MRSA USA300 [22]. After isonipecotic acid modification, compound **1a** was synthesized with an MIC value of 8 µg/mL against MRSA USA300. Compound **2a**, composed of ocotillol connected with succinic anhydride, had an MIC value of 16 µg/mL against RN4220. Additionally, when compound **1a** was combined with KAN, a synergistic antibacterial effect was observed against MRSA USA300 with an FICI of 0.28 [23,24] (Figure 2).

On the basis of the good anti-MRSA potency and synergistic antibacterial activity of the ocotillol-type ginsenosides [25], our group considered (20*S*, 24*S*)-ocotillol and its isomer (20*S*, 24*R*)-ocotillol as the lead compounds for structural modification. In our previous work, we synthesized different series of ocotillol-type derivatives, from which we identified certain SARs [22,23,26]. The antibacterial activity of ocotillol derivatives increases when the pharmacophore contains an amino group or is a hydrogen bond donor [27]. Additionally, studies have shown that nitrogen-containing heterocycles, such as triazoles, morpholines, succinimides, Fmoc-protected amino acids, and dansulfonyl chloride, have extensive and excellent pharmacological activities, especially in terms of their antibacterial properties [28,29,30,31]. It is well known that amino acids play important roles in various medical fields, particularly, in the antimicrobial [32]. Researchers have shown that Fmoc-conjugated amino acids (Fmoc-AA) have a broad range of antimicrobial effects, which linearly correlates with their surfactant property [33]. Therefore, in this research, to obtain compounds with good anti-drug-resistant bacterial potency or synergistic antibacterial activity and further explore the SARs, we synthesized two series of new 3-substituted ocotillol derivatives by introducing nitrogen-containing heterocycles and Fmoc-AA.

Because of the clinical misuse of antibiotics and the higher prevalence of bacteria in hospitals, hospital-acquired methicillin-resistant *Staphylococcus aureus* (HA-MRSA) spreads faster and is more resistant to marketed antibacterial drugs than common *Staphylococcus aureus* [34,35,36]. Therefore, we conducted antibacterial activity assays using *S. aureus* ATCC 29213, and HA-MRSA strains18–19 and 18–20 on the 18 compounds designed and synthesized in this work so that the experiment was representative of clinical conditions. Furthermore, compounds **20** and **21**, which had good antibacterial activity, were selected for evaluation of their synergistic antibacterial activity with KAN and CHL.

## 2. Results and Discussion

### 2.1. Chemistry

**Scheme 1:**
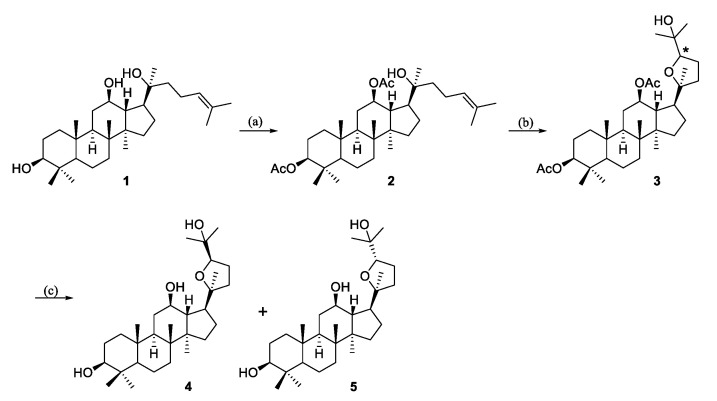
Synthetic route of ocotillol-type compounds **4** and **5**. Reagents and conditions: (**a**) Ac_2_O, DMAP, CH_2_Cl_2_, r.t.; (**b**) m-CPBA, CH_2_Cl_2_, r.t.; (**c**) KOH, CH_3_OH, 65 °C.

Compounds **4** and **5** were synthesized by a straightforward three-step procedure. The synthesis of ocotillol derivatives **4** and **5** followed previously reported procedures as shown in Scheme 1.

**Scheme 2:**
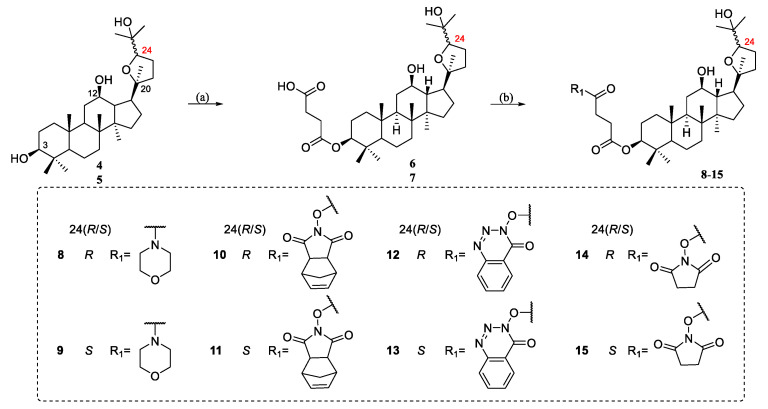
Synthetic route of ocotillol-type compounds **8**–**15**. Reagents and conditions: (**a**) Succinic anhydride, DMAP, CH_2_Cl_2_, r.t.; (**b**) RH, EDCI, CH_2_Cl_2_, r.t.

**Scheme 3:**
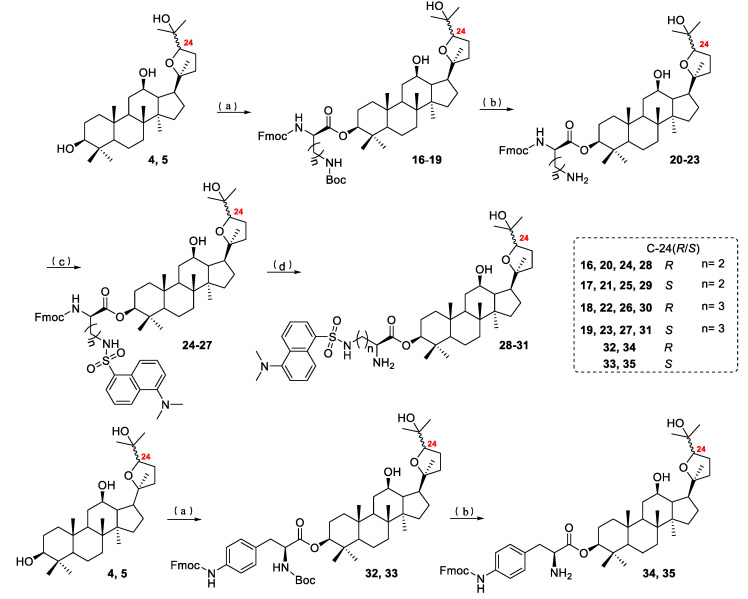
Synthetic route of ocotillol-type compounds **20**–**23**, **28**–**31**, **34**, and **35**. Reagents and conditions: (**a**) DMAP, EDCI, Boc-Fmoc-amino acid, CH_2_Cl_2_, r.t.; (**b**) TFA, CH_2_Cl_2_, r.t.; (**c**) DMAP, EDCI, Dansyl chloride, CH_2_Cl_2_, r.t.; (**d**) piperidine, CH_2_Cl_2_, r.t.

Ocotillol-type derivatives **8**–**15** were synthesized as shown in Scheme 2. Compound **6** or **7** was obtained by the reaction of compound **4** or **5** with succinic anhydride, then compounds **6** and **7** were coupled with different nitrogen-containing heterocycles in dry dichloromethane (DCM) as well as appropriate catalyst to produce compounds **8**–**15**. Ocotillol-type derivatives **20**–**23**, **28**–**31**, **34**, and **35** were synthesized as shown in Scheme 3. Compounds **20**–**23**, **28**–**31**, **34**, and **35** were obtained by introducing Fmoc or dansyl chloride through different diamine linkage chains on the basis of compound **4** or **5**.

### 2.2. Antibacterial Activity

The initial antibacterial activity results of the compounds against HA-MRSA 18–19, 18–20, and *S. aureus* ATCC 29213 are summarized in Table 1. Our previous research showed that ocotillol phthalic anhydride derivatives linked with *N*-containing heterocycles did not demonstrate antibacterial activity. Considering different functional groups and our previous work [24], succinic anhydride was used as a linker. However, the MICs of compounds **8**–**15** were greater than 128 μg/mL, thus the linker had no influence on the antibacterial activity.

Among the synthesized compounds, **20**–**31** inhibited the growth of HA-MRSAs 18–19, 18–20 and *S. aureus* ATCC 29213 in vitro, with MIC values of 1–64 µg/mL. Compound **21** using *L*-2,4-diaminobutyric acid hydrobromide as the linking chain to the Fmoc group showed better antibacterial activity, with an MIC value of 1 μg/mL, while compounds **22** and **23** had good antibacterial activities against HA-MRSA 18–20 with MICs of 2 µg/mL. Compared with compounds **20**–**23**, compounds **28**–**31** showed weaker antibacterial activity. Analysis of the structures of compounds **20**–**23** and **28**–**31** indicated that compounds **20**–**23**, which were linked to the Fmoc group, had better antibacterial activity than compounds **28**–**31**, which were linked to the dansyl chloride. The stereochemistry at C-24 dramatically affects the antibacterial activity, with the S-configuration preferred when C-3 hydroxyl is not substituted. However, the dramatic difference in antibacterial activity between the S-configuration and R-configuration caused by the stereochemistry at C-24 seemed to be overpowered by substitutions at C-3 hydroxyl [22,37]. Compounds **20**–**23** and **28**–**31**, which were substituted with Fmoc or dansyl chloride at the C-3 hydroxyl, showed no significant differences in activity among stereoisomers against the same drug-resistant strain.

**Figure 3:**
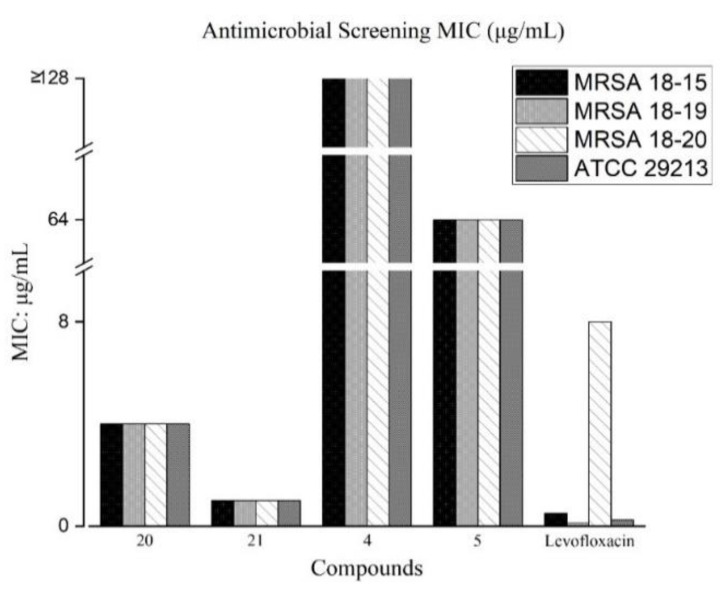
In vitro antibacterial activity of ocotillol-type derivatives (MIC: μg/mL).

Compound **20** and its isomer **21** were chosen for testing against HA-MRSA 18–15, 18–19, 18–20, and *S. aureus* ATCC 29213 (Figure 3). The result showed that compounds **20** and **21** have good antibacterial activity with an MIC of 4 and 1 μg/mL, respectively. It is suggested that the aromatic Fmoc and the length of the primary amine may play vital roles in the binding of the pharmacologically active compounds. On the basis of the results of the antibacterial activity and previous research in our laboratory, strain HA-MRSA 18–15 was chosen for assessment of synergistic antibacterial activity.

### 2.3. Synergistic Antibacterial Activity

Antibiotics with different phytochemicals are often used in combination to achieve a synergistic antibacterial effect in the clinical treatment of infections caused by drug-resistant bacteria [38]. According to the results in Table 2 and Table 3, compounds **20** and **21** possessed synergistic activity when combined with KAN or CHL (FICI < 0.5). FICI was defined as FICA + FICB = (MIC_A+B_/MIC_A_) + (MIC_A+B_/MIC_B_), and when the value of FICI was less than or equal to 0.5, compounds A and B were deemed to have a synergistic effect.

When compound **21** was used with CHL, the antibacterial activity of CHL against HA-MRSA 18–15 was enhanced from 8 to 1 μg/mL, and the FICI value was 0.375. When compounds **20** and **21** were used with KAN, the antibacterial activity of KAN against HA-MRSA 18–15 was significantly enhanced from 2 to 0.5 and 0.25 μg/mL, and the FICI value was 0.3125 and 0.375, respectively. This result showed that compounds **20** and **21** have good synergistic effects in the presence of KAN or CHL and compound **21** is expected to be developed as a synergistic antibacterial drug in the future, but the drug combination did not produce a bactericidal effect.

### 2.4. Structure–Activity Relationships (SARs)

**Figure 4:**
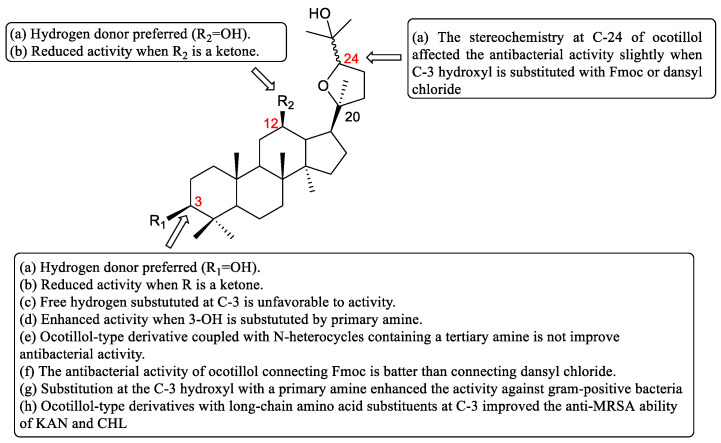
Structure–activity relationships (SARs) of ocotillol-type derivatives.

Based on previous data and our recent work, a more comprehensive structure–activity relationship for ocotillol-type derivatives was obtained as follows (Figure 4). (a) A hydrogen donor at C-3 and C-12 is preferred to maintain the antibacterial activity; (b) decreased antibacterial activity was observed when the functional groups at C-3 and C-12 were ketones; (c) ocotillol-type derivatives coupled with *N*-heterocycles containing a tertiary amine did not improve the antibacterial activity; (d) the stereochemistry at C-24 of ocotillol affected the antibacterial activity slightly when C-3 hydroxyl was substituted with Fmoc or dansyl chloride; (e) the antibacterial activity of ocotillol with a connecting Fmoc was better than those with a connecting dansyl chloride; (f) substitution at the C-3 hydroxyl with a primary amine enhanced the activity against Gram-positive bacteria; and(g) ocotillol-type derivatives with long-chain amino acid substituents at C-3 improved the anti-MRSA ability of KAN and CHL.

## 3. Materials and Methods

### 3.1. Chemical Reagents and Instruments

Most of the chemicals and solvents were of analytical grade and the solvents were purified and dried using standard methods. All the structures were verified by nuclear magnetic resonance (NMR), such as ^1^H-NMR, ^13^C-NMR, and high-resolution mass spectrometry (HR-MS). Spectra for all the compounds were obtained at 400 MHz for the ^1^H NMR spectra and at 100 MHz for the ^13^C NMR using a JNM-ECZ400S (JEOL Ltd., Japan) in CDCl_3_. HRMS was performed using a Q Exactive TM Orbitrap MS system (Thermo Scientific, USA) in CH_3_OH.

### 3.2. The Synthesis of Compounds ***8**–**15***

Compound **4** or **5** (500 mg, 1.05 mmol), 4-dimethylaminopyridine (383 mg, 3.14 mmol), and succinic anhydride (209 mg, 2.09 mmol) were placed in dry dichloromethane (10 mL), and after stirring at room temperature for 6 h, the resulting mixture was washed with 10% HCl, water, and brine, dried over anhydrous Na_2_SO_4_, filtered, and evaporated. The white powder intermediate **6** or **7** (542 mg, 0.94 mmol) was obtained.

A nitrogen-containing heterocycle (morpholine, 3,4-dihydro-3-hydroxy-4-oxo-1,2,3-benzotriazine, *N*-hydroxy-5-norborene-2,3-dicarboximide, or 1-hydroxy-5-pyrrolidinedione) (0.19 mmol), and 1-ethyl-3-(3-dimethylaminopropyl) carbodiimide hydrochloride (EDCI) (30 mg, 0.14 mmol) was combined with compound **6** (60 mg, 0.10 mmol) in anhydrous dichloromethane (5 mL), and the resulting mixture was stirred for 6 h at room temperature. The organic solution was washed with water and brine, and dried over anhydrous Na_2_SO_4_. The dichloromethane was evaporated in vacuo and the residue was purified by column chromatography (7:1–3:1 petroleum ether: ethyl acetate) to give the desired products **8**–**15**.

(20S, 24R)-Epoxy-3β-*O*-[2-(1H-morpholin-1-carbonyl) carboxy propionyl]-dammarane-12β, 25-diol (**8**)

White solid (yield 73.2%); m.p. 114.5~115.3 °C; [α]D 20 = 41.342 (c = 0.1, MeOH:CHCl_3_ = 3:1); ^1^H-NMR (CDCl_3_, 400 MHz) δ (ppm): 4.46 (dd, *J* = 10.7 Hz, 5.6 Hz, 1H, -OCH-), 3.82 (dd, *J* = 8.8 Hz, 6.7 Hz, 1H, -OCH-), 3.71-3.62 (m, 4H, -CH_2_-×2), 3.59 (d, *J* = 5.1 Hz, 2H, -CH_2_-), 3.47 (q, *J* = 4.4 Hz, 3H, -CH_2_-, -OCH-), 2.64 (d, *J* = 5.2 Hz, 2H, -CH_2_-), 2.59 (d, *J* = 5.7 Hz, 2H, -CH_2_-), 1.25 (s, 3H, -CH_3_), 1.24 (s, 3H, -CH_3_), 1.07 (s, 3H, -CH_3_), 0.96 (s, 3H, -CH_3_), 0.87 (s, 3H, -CH_3_), 0.85 (s, 3H, -CH_3_), 0.83 (s, 6H, -CH_3_×2). ^13^C-NMR (CDCl_3_, 100 MHz) δ (ppm): 172.78, 170.05, 86.60, 85.50, 81.09, 71.03, 70.19, 66.95, 66.62, 56.18, 52.10, 50.50, 49.47, 48.04, 45.80, 42.15, 39.85, 38.70, 38.01, 37.15, 34.84, 32.69, 31.42, 31.28, 29.66, 28.67, 28.04, 27.67, 26.21, 25.08, 23.75, 18.24, 16.55, 15.47. HR-MS (ESI) *m*/*z*: calcd. for C_38_H_63_NO_7_ [M+H]^+^: 646.46773, found: 646.46735.

(20*S*, 24*S*)-Epoxy-3*β*-*O*-[2-(1*H*-morpholin-oxo-carbonyl) carboxy propionyl]-dammarane-12*β*, 25-diol (**9**).

White solid (yield 70.3%); m.p. 98.5~99.3 °C; [α${]}_{20}^{D}$ = 39.682 (c = 0.1, MeOH:CHCl_3_ = 3:1); ^1^H-NMR (CDCl_3_, 400 MHz) δ (ppm): 4.46 (dd, *J* = 10.6, 5.8 Hz, 1H, -OCH-), 3.84 (dd, *J* = 10.8, 5.4 Hz, 1H, -OCH-), 3.64 (d, *J* = 5.1 Hz, 4H, -CH_2_-×2), 3.58 (d, *J* = 5.1 Hz, 2H, -CH_2_-), 3.50 (dd, *J* = 10.4, 4.8 Hz, 1H, -OCH-), 3.47 (d, *J* = 5.0 Hz, 2H, -CH_2_-), 2.64 (dd, *J* = 8.2, 6.0 Hz, 2H, -CH_2_-), 2.63 – 2.55 (m, 2H, -CH_2_-), 1.24 (s, 3H, -CH_3_), 1.20 (s, 3H, -CH_3_), 1.07 (s, 3H, -CH_3_), 0.98 (s, 3H, -CH_3_), 0.88 (s, 6H, -CH_3_×2), 0.82 (s, 6H, -CH_3_×2). ^13^C-NMR (CDCl_3_, 100 MHz) δ (ppm): 172.74, 170.02, 87.45, 87.19, 81.07, 70.55, 70.09, 66.94, 66.60, 56.12, 52.23, 50.21, 49.00, 48.88, 45.77, 42.14, 39.85, 38.64, 38.02, 37.15, 34.77, 32.28, 31.77, 29.65, 28.95, 28.61, 28.06, 27.90, 25.13, 24.33, 23.75, 18.25, 17.85, 16.58, 16.40, 15.54. HR-MS (ESI) *m*/*z*: calcd. for C_38_H_63_NO_7_ [M+H]^+^: 646.46773, found: 646.46692.

(20*S*, 24*R*)-Epoxy-3*β*-*O*-[2-(*N*-hydroxy-5-norbornene-2,3-dicarboximide-oxo-carbonyl) carboxy propionyl]-dammarane-12*β*, 25-diol (**10**).

White solid (yield 82.3%); m.p. 105.9~106.3 °C; [α${]}_{20}^{D}$ = 49.351 (c = 0.1, MeOH:CHCl_3_ = 3:1); ^1^H-NMR (CDCl_3_, 400 MHz) δ (ppm): δ 6.18 (s, 2H, -CH=CH-), 4.57 – 4.46 (m, 1H, -OCH-), 3.84 (dd, *J* = 8.8, 6.5 Hz, 1H, -OCH-), 3.51 (td, *J* = 10.5, 4.6 Hz, 1H, -OCH-), 3.44 (s, 2H, -CH-×2), 3.31 (s, 2H, -CH-×2), 2.89 (t, *J* = 6.1 Hz, 2H, -CH_2_-), 2.70 (t, *J* = 6.8 Hz, 2H, -CH_2_-), 1.27 (s, 3H, -CH_3_), 1.27 (s, 3H, -CH_3_), 1.09 (s, 3H, -CH_3_), 0.98 (s, 3H, -CH_3_), 0.90 (s, 3H, -CH_3_), 0.88 (s, 3H, -CH_3_), 0.84 (s, 3H, -CH_3_), 0.82 (s, 3H, -CH_3_). ^13^C-NMR (CDCl_3_, 100 MHz) δ (ppm): 170.79, 169.90, 134.77, 86.59, 85.47, 81.77, 71.02, 70.21, 56.12, 51.88, 50.47, 49.45, 48.03, 39.83, 38.68, 37.97, 37.13, 34.82, 32.68, 31.40, 31.26, 29.17, 28.66, 28.02, 27.65, 26.20, 25.07, 24.09, 23.63, 21.12, 18.22, 17.44, 16.51, 15.45. HR-MS (ESI) *m*/*z*: calcd. for C_43_H_63_NO_9_ [M+H]^+^: 738.45756, found: 738.45691.

(20*S*, 24*S*)-Epoxy-3*β*-*O*-[2-(*N*-hydroxy-5-norbornene-2,3-dicarboximide-oxo-carbonyl) carboxy propionyl]-dammarane-12*β*, 25-diol (**11**).

White solid (yield 79.5%); m.p. 101.8~102.3 °C; [α]D 20 = 57.355 (c = 0.1, MeOH:CHCl_3_ = 3:1); ^1^H-NMR (CDCl_3_, 400 MHz) δ (ppm): 6.16 (s, 2H, -CH=CH-), 4.54 – 4.46 (m, 1H, -OCH-), 3.86 (dd, *J* = 10.6, 5.1 Hz, 1H, -OCH-), 3.56 – 3.47 (m, 1H, -OCH-), 3.42 (s, 2H, -CH-×2), 3.29 (s, 2H, -CH-×2), 2.87 (t, *J* = 6.6 Hz, 2H, -CH_2_-), 2.68 (t, *J* = 7.3 Hz, 2H, -CH_2_-), 1.25 (s, 3H, -CH_3_), 1.21 (s, 3H, -CH_3_), 1.08 (s, 3H, -CH_3_), 0.99 (s, 3H, -CH_3_), 0.89 (s, 6H, -CH_3_×2), 0.83 (s, 3H, -CH_3_), 0.81 (s, 3H, -CH_3_). ^13^C-NMR (CDCl_3_, 100 MHz) δ (ppm): 170.73, 169.88, 134.85, 87.48, 87.24, 81.75, 70.59, 70.15, 56.09, 52.24, 50.20, 48.98, 48.86, 44.80, 39.84, 37.99, 37.15, 34.76, 32.29, 31.72, 29.19, 28.95, 28.62, 28.07, 28.04, 26.41, 25.15, 24.28, 21.13, 18.25, 17.84, 16.54, 16.39, 15.54. HR-MS (ESI) *m*/*z*: calcd. for C_43_H_63_NO_9_ [M+H]^+^: 738.45756, found: 738.45703.

(20*S*, 24*R*)-Epoxy-3*β*-*O*-[2-(3-hydroxy-1,2,3-benzotriazin-4(3H)-oxo-carbonyl) carboxy propionyl]-dammarane-12*β*, 25-diol (**12**).

Yellow solid (yield 84.1%); m.p. 154.5~154.9 °C; [α${]}_{20}^{D}$ = 50.020 (c = 0.1, MeOH:CHCl_3_ =3:1);^1^H-NMR (CDCl_3_, 400 MHz) δ (ppm): 8.39 – 8.35 (m, 1H, Ar-H), 8.22 (dt, *J* = 8.1, 0.5 Hz, 1H, Ar-H), 8.00 (ddd, *J* = 8.6, 7.3, 1.4 Hz, 1H, Ar-H), 7.84 (ddd, *J* = 8.4, 7.3, 0.8 Hz, 1H, Ar-H), 4.48 (dd, *J* = 10.6, 5.6 Hz, 1H, -OCH-), 3.83 (dd, *J* = 8.5, 6.7 Hz, 1H, -OCH-), 3.54 – 3.49 (m, 1H, -OCH-), 2.62 (s, 4H, -CH_2_-×2), 1.24 (s, 3H, -CH_3_), 1.21 (s, 3H, -CH_3_), 1.08 (s, 3H, -CH_3_), 0.97 (s, 6H, -CH_3_×2), 0.88 (s, 3H, -CH_3_), 0.87 (s, 3H, -CH_3_), 0.83 (s, 3H, -CH_3_). ^13^C-NMR (CDCl_3_, 100 MHz) δ (ppm): 170.88, 168.71, 150.27, 144.41, 135.56, 132.87, 129.12, 125.88, 122.33, 86.61, 85.48, 81.89, 71.04, 70.21, 60.53, 56.11, 52.08, 51.97, 50.45, 49.45, 48.00, 39.80, 38.65, 37.99, 37.96, 37.12, 34.79, 32.71, 31.29, 29.15, 28.68, 28.02, 27.74, 26.77, 26.22, 25.11, 21.21, 18.28, 16.58, 15.44. HR-MS (ESI) *m*/*z*: calcd. for C_41_H_59_N_3_O_8_ [M+H]^+^: 722.43749, found: 722.43677.

(20*S*, 24*S*)-Epoxy-3*β*-*O*-[2-(3-hydroxy-1,2,3-benzotriazin-4(3H)-oxo-carbonyl) carboxy propionyl]-dammarane-12*β*, 25-diol (**13**).

Yellow solid (yield 69.3%); m.p. 149.5~150.1 °C; [α${]}_{20}^{D}$ = 31.346 (c = 0.1, MeOH:CHCl_3_ = 3:1); ^1^H-NMR (CDCl_3_, 400 MHz) δ (ppm): 8.34 (dd, *J* = 8.0, 1.4 Hz, 1H, Ar-H), 8.23 – 8.18 (m, 1H, Ar-H), 7.98 (ddd, *J* = 8.7, 7.3, 1.4 Hz, 1H, Ar-H), 7.82 (ddd, *J* = 8.4, 7.4, 1.1 Hz, 1H, Ar-H), 4.56 – 4.50 (m, 1H, -OCH-), 3.85 (dd, *J* = 10.8, 5.3 Hz, 1H, -OCH-), 3.51 (td, *J* = 10.3, 4.6 Hz, 1H, -OCH-), 2.86 – 2.76 (m, 2H, -CH_2_-), 2.65 – 2.55 (m, 2H, -CH_2_-), 1.24 (s, 3H, -CH_3_), 1.20 (s, 3H, -CH_3_), 1.07 (s, 3H, -CH_3_), 0.98 (s, 3H, -CH_3_), 0.88 (s, 6H, -CH_3_×2), 0.85 (s, 3H, -CH_3_), 0.83 (s, 3H, -CH_3_). ^13^C-NMR (CDCl_3_, 100 MHz) δ (ppm): 170.82, 168.68, 150.25, 144.40, 135.52, 132.83, 129.10, 125.85, 122.32, 87.47, 87.23, 81.88, 60.49, 56.07, 52.23, 50.18, 48.93, 48.81, 39.81, 38.61, 37.99, 37.13, 34.73, 32.29, 31.69, 29.16, 28.94, 28.61, 28.07, 26.78, 25.17, 24.24, 18.24, 17.85, 16.58, 16.40, 15.52. HR-MS (ESI) *m*/*z*: calcd. for C_41_H_59_N_3_O_8_ [M+H]^+^: 722.43749, found: 722.43309.

(20*S*, 24*R*)-Epoxy-3*β*-*O*-[2-(N-hydroxy-succinimide-oxo-carbonyl) carboxy propionyl]-damma-rane-12*β*, 25-diol (**14**).

White solid (yield 82.5%); m.p. 115.5~116.3 °C. [α]D 20 = 97.039 (c = 0.1, MeOH:CHCl_3_ = 3:1); ^1^H-NMR (CDCl_3_, 400 MHz) δ (ppm): 4.55 – 4.45 (m, 1H, -OCH-), 3.83 (dd, *J* = 8.8, 6.7 Hz, 1H, -OCH-), 3.49 (td, *J* = 10.5, 4.5 Hz, 1H, -OCH-), 2.94 (td, *J* = 7.1, 6.7, 2.1 Hz, 2H, -CH_2_-), 2.82 (s, 4H, -CH_2_-×2), 2.75 – 2.67 (m, 2H, -CH_2_-), 1.26 (s, 3H, -CH_3_), 1.25 (s, 3H, -CH_3_), 1.08 (s, 3H, -CH_3_), 0.96 (s, 3H, -CH_3_), 0.88 (s, 3H, -CH_3_), 0.86 (s, 3H, -CH_3_), 0.84 (s, 3H, -CH_3_), 0.82 (s, 3H, -CH_3_). ^13^C-NMR (CDCl_3_, 100 MHz) δ (ppm): 170.71, 168.92, 167.80, 86.60, 85.48, 81.85, 71.04, 70.22, 56.14, 52.10, 50.49, 49.46, 48.04, 39.84, 38.69, 37.99, 37.15, 34.83, 32.69, 31.40, 31.27, 30.65, 29.15, 28.67, 27.98, 27.66, 26.44, 26.21, 25.64, 25.08, 23.66, 18.24, 16.53, 15.47. HR-MS (ESI) *m*/*z*: calcd. for C_38_H_59_NO_9_ [M+H]^+^: 674.42626, found: 674.42621.

(20*S*, 24*S*)-Epoxy-3*β*-*O*-[2-(N-hydroxy-succinimide-oxo-carbonyl) carboxy propionyl]-damma-rane-12*β*, 25-diol (**15**).

White solid (yield 78.3%); m.p. 110.2~110.9 °C. [α]D 20 = 42.692 (c = 0.1, MeOH:CHCl_3_ = 3:1); ^1^H-NMR (CDCl_3_, 400 MHz) δ (ppm): 4.54 – 4.49 (m, 1H, -OCH-), 3.85 (dd, *J* = 10.8, 5.4 Hz, 1H, -OCH-), 3.51 (td, *J* = 10.4, 4.9 Hz, 1H, -OCH-), 2.94 (t, *J* = 7.4 Hz, 2H, -CH_2_-), 2.81 (s, 4H, -CH_2_-×2), 2.72 (t, *J* = 6.8 Hz, 2H, -CH_2_-), 1.25 (s, 3H, -CH_3_), 1.21 (s, 3H, -CH_3_), 1.08 (s, 3H, -CH_3_), 0.99 (s, 3H, -CH_3_), 0.89 (s, 6H, -CH_3_×2), 0.84 (s, 3H, -CH_3_), 0.82 (s, 3H, -CH_3_). ^13^C-NMR (CDCl_3_, 100 MHz) δ (ppm): 170.69, 168.94, 167.81, 87.46, 87.22, 81.84, 70.55, 70.13, 60.47, 56.09, 52.23, 50.20, 48.89, 39.84, 38.63, 37.99, 37.15, 34.76, 32.28, 31.70, 29.14, 28.96, 28.62, 28.06, 26.44, 25.64, 25.14, 24.31, 23.67, 18.25, 17.84, 16.55, 16.39, 15.54. HR-MS (ESI) *m*/*z*: calcd. for C_38_H_59_NO_9_ [M+H]^+^: 674.42626, found: 674.42548.

### 3.3. The Synthesis of Compounds ***20**–**23***, ***28**–**31***, ***34***, and ***35***

Compound **4** or **5** (80 mg, 0.17 mmol) was added to anhydrous dichloromethane (4 mL), then a Boc-Fmoc-protected amino acid (0.25 mmol), DMAP (56 mg, 0.50 mmol), and EDCI (96 mg, 0.50 mmol) were added slowly. After stirring at room temperature for 3 h, the resulting mixture was washed with 10% HCl, water, and brine, and dried over anhydrous Na_2_SO_4_. The dichloromethane was evaporated in vacuo to obtain the intermediates **16**–**19**, **32**, and **33**. Trifluoroacetic acid (1 mL, 13.43 mmol) was added slowly to a solution of each intermediate **16**–**19**, **32**, and **33** (0.17 mmol) in anhydrous dichloromethane (4 mL). After stirring at room temperature for 2 h, the resulting mixture was filtered and evaporated to obtain the target compounds **20**–**23**, **34**, and **35**. For compounds **20**–**23**, each compound (0.17 mmol), dansyl chloride (55 mg, 0.20 mmol), and *N*, *N*-diisopropylethylamine (106 μL, 0.65 mmol) were combined in anhydrous dichloromethane (4 mL). After stirring at room temperature for 2 h, the resulting mixture was washed with 5% HCl, water, and brine, dried over in anhydrous Na_2_SO_4_, filtered, and evaporated to provide intermediates **24**–**27** (Scheme 3). Compounds **28**–**31** were synthesized by adding intermediates **24**–**27** (0.17 mmol) and piperidine (1 mL, 10.88 mmol) to anhydrous dichloromethane (4 mL). After stirring at room temperature for 4 h, the resulting mixture was washed with water and brine, dried over anhydrous Na_2_SO_4_, filtered, and evaporated. The residue was purified over silica gel with chloroform: methanol (100:1–50:1) to provide compounds **28**–**31**.

#### 3.3.1. (20S, 24R)-Epoxy-3β-*O*-[2-(*N’*-Fmoc)-5-amino butyryl]-dammarane-12β, 25-diol (**20**)

White solid (yield 77.4%); m.p. 174.2~174.7 °C; [α${]}_{20}^{D}$ = 108.370 (c = 0.1, MeOH:CHCl^3^ = 3:1); ^1^H-NMR (CDCl_3_, 400 MHz) δ (ppm): 8.27 (s, 2H, -NH_2_), 7.70 (d, *J* = 7.6 Hz, 2H, Ar-H×2), 7.55 (d, *J* = 6.6 Hz, 2H, Ar-H×2), 7.34 (t, *J* = 7.4 Hz, 2H, Ar-H×2), 7.26 (t, *J* = 7.5 Hz, 2H, Ar-H×2), 6.20 (d, *J* = 7.4 Hz, 1H, -NH-), 4.47 (d, *J* = 7.3 Hz, 1H, -OCH-), 4.32 (d, *J* = 6.5 Hz, 3H, -OCH_2_-, -NCH-), 4.14 (t, *J* = 6.8 Hz, 1H, -CH-), 3.81 (t, *J* = 7.6 Hz, 1H, -OCH-), 3.60 − 3.47 (m, 1H, -OCH-), 3.04 (d, *J* = 43.0 Hz, 2H, -NCH_2_-), 2.23 (d, *J* = 55.9 Hz, 2H, -CH_2_-), 1.26 (s, 3H, -CH_3_), 1.24 (s, 3H, -CH_3_), 1.22 (s, 3H, -CH_3_), 1.09 (s, 3H, -CH_3_), 0.94 (s, 3H, -CH_3_), 0.86 (s, 3H, -CH_3_), 0.82 (s, 3H, -CH_3_), 0.79 (s, 3H, -CH_3_). ^13^C-NMR (CDCl_3_, 100 MHz) δ (ppm): 171.18, 157.09, 143.75, 141.32, 132.50, 131.01, 128.89, 127.82, 127.21, 125.20, 120.04, 86.52, 85.24, 83.25, 71.13, 70.42, 68.24, 67.47, 56.08, 52.10, 51.59, 50.35, 49.28, 48.07, 47.08, 39.78, 38.79, 38.48, 37.95, 37.06, 34.75, 32.52, 32.03, 31.36, 31.11, 29.80, 29.01, 28.01, 27.48, 26.10, 25.09, 23.81, 23.35, 23.09, 22.80, 18.17, 16.25, 15.47. HR-MS (ESI) *m*/*z*: calcd. for C_49_H_70_N_2_O_7_ [M+H]^+^: 799.52558, found: 799.52283.

#### 3.3.2. (20S, 24S)-Epoxy-3β-*O*-[2-(*N’*-Fmoc)-5-amino butyryl]-dammarane-12β, 25-diol (**21**)

White solid (yield 70.1%); m.p. 191.1~191.5 °C; [α${]}_{20}^{D}$ = 48.686 (c = 0.1, MeOH:CHCl_3_ = 3:1); ^1^H-NMR (CDCl_3_, 400 MHz) δ (ppm): 8.24 (s, 2H, -NH_2_), 7.69 (d, *J* = 7.5 Hz, 2H, Ar-H×2), 7.54 (d, *J* = 5.6 Hz, 2H, Ar-H×2), 7.34 (t, *J* = 7.4 Hz, 2H, Ar-H×2), 7.25 (t, *J* = 7.4 Hz, 3H, Ar-H×2), 6.13 (d, *J* = 7.5 Hz, 1H, -NH-), 4.55 − 4.43 (m, 1H, -OCH-), 4.40 − 4.26 (m, 3H, -OCH_2_-, -NCH-), 4.13 (t, *J* = 6.9 Hz, 1H, -CH-), 3.92 − 3.79 (m, 1H, -OCH-), 3.50 (s, 1H, -OCH-), 3.02 (d, *J* = 55.7 Hz, 2H, -NCH_2_-), 2.26 (d, *J* = 41.9 Hz, 2H, -CH_2_-), 1.24 (s, 3H, -CH_3_), 1.23 (s, 3H, -CH_3_), 1.18 (s, 3H, -CH_3_), 1.10 (s, 3H, -CH_3_), 0.96 (s, 3H, -CH_3_), 0.85 (s, 3H, -CH*3*), 0.84 (s, 3H, -CH*3*), 0.79 (s, 3H, -CH_3_). 13C-NMR (CDCl3, 100 MHz) δ (ppm): 171.13, 157.02, 143.70, 141.32, 127.81, 127.22, 125.29, 125.27, 125.17, 120.03, 87.66, 87.19, 83.26, 70.66, 67.45, 64.51, 61.00, 55.99, 52.19, 51.54, 50.15, 48.87, 48.71, 47.07, 46.29, 39.76, 39.18, 38.47, 37.92, 37.04, 36.61, 34.67, 32.36, 32.03, 31.69, 30.22, 29.80, 29.47, 28.91, 28.55, 27.99, 25.53, 23.75, 23.46, 22.80, 18.19, 16.28, 15.47. HR-MS (ESI) *m*/*z*: calcd. for C_49_H_70_N_2_O_7_ [M+H]^+^: 799.52558, found: 799.52307.

#### 3.3.3. (20S, 24R)-Epoxy-3β-*O*-[2-(*N’*-Fmoc)-6-amino ornithyl]-dammarane-12β, 25-diol (**22**)

White solid (yield 69.9%); m.p. 183.5~184.1 °C; [α${]}_{20}^{D}$ = 34.345 (c = 0.1, MeOH:CHCl_3_ = 3:1); ^1^H-NMR (CDCl_3_, 400 MHz) δ (ppm): 8.01 (s, 2H, -NH_2_), 7.69 (d, *J* = 7.5 Hz, 2H, Ar-H×2), 7.54 (t, *J* = 7.0 Hz, 2H, Ar-H×2), 7.32 (t, *J* = 7.5 Hz, 2H, Ar-H×2), 7.24 (d, *J* = 5.7 Hz, 2H, Ar-H×2), 5.84 (d, *J* = 8.0 Hz, 1H, -NH-), 4.50 − 4.38 (m, 1H, -OCH-), 4.27 (d, *J* = 6.8 Hz, 3H, -OCH_2_-, -NCH-), 4.12 (t, *J* = 7.0 Hz, 1H, -CH-), 3.86 − 3.77 (m, 1H, -OCH-), 3.48 (dd, J = 10.2, 4.1 Hz, 1H, -OCH-), 2.95 (s, 2H, -NCH_2_-), 1.25 (s, 3H, -CH_3_), 1.24 (s, 6H, -CH3×2), 1.08 (s, 3H, -CH_3_), 0.93 (s, 3H, -CH_3_), 0.83 (s, 3H, -CH_3_), 0.79 (s, 3H, -CH_3_), 0.75 (s, 3H, -CH_3_). ^13^C-NMR (CDCl_3_, 100 MHz) δ (ppm): 171.81, 156.55, 143.95, 143.72, 141.28, 127.78, 127.18, 125.31, 125.15, 120.01, 86.58, 85.40, 82.84, 71.00, 70.29, 55.98, 52.05, 50.39, 49.38, 48.02, 47.05, 39.79, 39.36, 37.05, 34.75, 32.65, 32.01, 31.40, 31.23, 29.78, 29.44, 28.66, 27.98, 27.59, 26.15, 23.74, 23.62, 22.77, 18.18, 16.49, 16.36, 15.43. HR-MS (ESI) *m*/*z*: calcd. for C_50_H_72_N_2_O_7_ [M+H]^+^: 813.54123, found: 813.54254.

#### 3.3.4. (20S, 24S)-Epoxy-3β-*O*-[2-(*N’*-Fmoc)-6-amino ornithyl]-dammarane-12β, 25-diol (**23**)

White solid (yield 67.9%); m.p. 177.2~177.9 °C; [α${]}_{20}^{D}$ = 96.034 (c = 0.1, MeOH:CHCl_3_ = 3:1); ^1^H-NMR (CDCl3, 400 MHz) δ (ppm): 8.07 (s, 2H, -NH_2_), 7.69 (d, *J* = 7.5 Hz, 2H, Ar-H×2), 7.55 (t, *J* = 6.3 Hz, 2H, Ar-H×2), 7.33 (t, *J* = 7.5 Hz, 2H, Ar-H×2), 7.24 (t, *J* = 7.4 Hz, 2H, Ar-H×2), 5.83 (d, *J* = 8.0 Hz, 1H, -NH-), 4.47 (t, *J* = 8.7 Hz, 1H, -OCH-), 4.28 (d, *J* = 6.6 Hz, 3H, -OCH_2_-, -NCH-), 4.13 (t, *J* = 7.1 Hz, 1H, -CH-), 3.90 − 3.81 (m, 1H, -OCH-), 3.54 − 3.44 (m, 1H, -OCH-), 2.97 (s, 2H, -NCH_2_-), 1.24 (s, 3H, -CH_3_), 1.19 (s, 3H, -CH_3_), 1.09 (s, 3H, -CH_3_), 0.96 (s, 3H, -CH_3_), 0.85 (s, 3H, -CH_3_), 0.84 (s, 3H, -CH_3_), 0.77 (s, 3H, -CH_3_), 0.76 (s, 3H, -CH3). ^13^C-NMR (CDCl_3_, 100 MHz) δ (ppm): 171.76, 156.54, 143.95, 143.74, 141.29, 127.78, 127.18, 125.32, 125.18, 120.01, 87.60, 87.22, 82.83, 70.66, 70.43, 67.41, 58.42, 55.94, 53.85, 52.21, 50.17, 48.93, 48.75, 47.06, 39.77, 39.38, 38.52, 37.96, 37.07, 34.69, 32.32, 31.76, 29.78, 28.90, 28.56, 28.01, 27.84, 25.41, 24.01, 18.17, 16.53, 16.31, 15.49. HR-MS (ESI) *m*/*z*: calcd. for C_50_H_72_N_2_O_7_ [M+H]^+^: 813.54123, found: 813.54169.

#### 3.3.5. (20S, 24R)-Epoxy-3β-*O*-[2-amino-(1-dansylamino)-5-dimethyl amino butyr-yl]-dammarane-12β, 25-diol (**28**)

Yellow solid (yield 72.2%); m.p. 202.0~202.7 °C; [α${]}_{20}^{D}$ = 51.689 (c = 0.1, MeOH:CHCl_3_ = 3:1); ^1^H-NMR (CDCl3, 400 MHz) δ (ppm): 8.50 (dt, *J* = 8.5, 1.0 Hz, 1H, Ar-H), 8.27 (d, *J* = 8.7 Hz, 1H, Ar-H), 8.22 (dd, *J* = 7.3, 1.3 Hz, 1H, Ar-H), 7.51 (ddd, *J* = 14.7, 8.6, 7.4 Hz, 2H, Ar-H×2), 7.16 (dd, *J* = 7.6, 0.7 Hz, 1H, Ar-H), 5.60 (s, 1H, -OH), 4.36 (dd, *J* = 10.6, 5.9 Hz, 1H, -OCH-), 3.82 (dd, *J* = 8.8, 6.5 Hz, 1H, -OCH-), 3.49 − 3.42 (m, 1H, -OCH-), 3.30 (dd, *J* = 9.6, 3.5 Hz, 1H, -NH_2_CH-), 3.14 (dd, *J* = 11.7, 7.4 Hz, 1H, -NCH_2_-), 3.01 − 2.91 (m, 1H, -NCH_2_-), 2.87 (s, 6H, -CH3×2), 1.25 (s, 3H, -CH_3_), 1.24 (s, 3H, -CH_3_), 1.07 (s, 3H, -CH_3_), 0.94 (s, 3H, -CH_3_), 0.86 (s, 3H, -CH_3_), 0.81 (s, 3H, -CH_3_), 0.73 (s, 3H, -CH_3_), 0.72 (s, 3H, -CH_3_). ^13^C-NMR (CDCl_3_, 100 MHz) δ (ppm): 174.52, 152.04, 134.72, 130.34, 129.95, 129.72, 128.24, 129.64, 123.27, 118.97, 115.20, 86.59, 85.49, 81.96, 70.98, 70.19, 56.03, 54.36, 52.06, 50.44, 49.45, 48.00, 45.51, 42.32, 39.80, 38.54, 37.92, 37.08, 34.75, 32.70, 32.36, 31.40, 31.28, 29.79, 28.66, 28.03, 28.01, 27.71, 26.22, 25.09, 23.56, 18.24, 16.51, 15.44. HR-MS (ESI) *m*/*z*: calcd. for C_46_H_71_N_3_O_7_S [M+H]^+^: 810.50855, found: 810.50635.

#### 3.3.6. (20S, 24S)-Epoxy-3β-*O*-[2-amino-(1-dansylamino)-5-dimethyl amino butyr-yl]-dammarane-12β, 25-diol (**29**)

Yellow solid (yield 69.6%); m.p. 210.5~211.3 °C; [α${]}_{20}^{D}$ = 84.703 (c = 0.1, MeOH:CHCl_3_ = 3:1); ^1^H-NMR (CDCl3, 400 MHz) δ (ppm): 8.51 (d, *J* = 8.4 Hz, 1H, Ar-H), 8.27 (d, *J* = 8.6 Hz, 1H, Ar-H), 8.22 (d, *J* = 7.3 Hz, 1H, Ar-H), 7.56 (s, 1H, Ar-H), 7.52 − 7.47 (m, 1H, Ar-H), 7.17 (d, *J* = 7.5 Hz, 1H, Ar-H), 5.75 (s, 1H, -OH), 4.38 (dd, *J* = 10.8, 5.8 Hz, 1H, -OCH-), 3.85 (dd, *J* = 10.9, 5.3 Hz, 1H, -OCH-), 3.50 (td, *J* = 9.8, 4.7 Hz, 1H, -OCH-), 3.30 (dd, *J* = 9.7, 3.5 Hz, 1H, -NH_2_CH-), 3.20 − 3.12 (m, 1H, -NCH_2_-), 2.97 (dd, *J* = 8.9, 4.2 Hz, 1H, -NCH_2_-), 2.87 (s, 6H, -CH3×2), 1.26 (s, 3H, -CH_3_), 1.23 (s, 3H, -CH_3_), 1.08 (s, 3H, -CH_3_), 0.98 (s, 3H, -CH_3_), 0.88 (s, 3H, -CH_3_), 0.86 (s, 3H, -CH_3_), 0.75 (s, 3H, -CH_3_), 0.74 (s, 3H, -CH_3_). 13C-NMR (CDCl_3_, 100 MHz) δ (ppm): 174.53, 152.06, 135.11, 130.34, 129.97, 129.73, 129.65, 128.25, 123.27, 118.98, 115.21, 87.47, 87.23, 81.98, 70.52, 70.11, 56.00, 54.44, 52.22, 50.18, 48.96, 48.86, 45.52, 39.81, 38.22, 37.93, 37.11, 34.70, 32.28, 31.69, 31.33, 29.80, 28.97, 28.61, 28.15, 27.30, 25.12, 24.30, 23.60, 18.20, 17.84, 16.54, 16.40, 15.52. HR-MS (ESI) *m*/*z*: calcd. for C_46_H_71_N_3_O_7_S [M+H]^+^: 810.50855, found: 810.50653.

#### 3.3.7. (20S, 24R)-Epoxy-3β-*O*-[2-amino-(1-dansylamino)-5-dimethyl amino val-eryl]-dammarane-12β, 25-diol (**30**)

Yellow solid (yield 65.1%); m.p. 192.5~192.9 °C; [α${]}_{20}^{D}$ = 59.357 (c = 0.1, MeOH:CHCl_3_ = 3:1); ^1^H-NMR (CDCl3, 400 MHz) δ (ppm): 8.50 (d, *J* = 8.5 Hz, 1H, Ar-H), 8.29 (d, *J* = 8.7 Hz, 1H, Ar-H), 8.21 (dd, *J* = 7.3, 1.3 Hz, 1H, Ar-H), 7.55 − 7.50 (m, 1H, Ar-H), 7.51 − 7.46 (m, 1H, Ar-H), 7.15 (d, *J* = 7.5 Hz, 1H, Ar-H), 5.57 (s, 1H. -OH), 4.47 − 4.39 (m, 1H, -OCH-), 3.82 (dd, *J* = 8.8, 6.6 Hz, 1H, -OCH-), 3.49 (td, *J* = 10.5, 4.6 Hz, 1H, -OCH-), 3.21 (dd, *J* = 8.6, 4.1 Hz, 1H, -NH_2_CH-), 2.99 − 2.91 (m, 1H, -NCH_2_-), 2.86 (s, 6H, -CH3×2), 2.81 (ddd, *J* = 12.7, 7.5, 5.4 Hz, 1H, -NCH_2_-), 1.25 (s, 3H, -CH_3_), 1.24 (s, 3H, -CH_3_), 1.07 (s, 3H, -CH_3_), 0.96 (s, 3H, -CH_3_), 0.87 (s, 3H, -CH_3_), 0.84 (s, 3H, -CH_3_), 0.77 (s, 3H, -CH_3_), 0.74 (s, 3H, -CH_3_). ^13^C-NMR (CDCl_3_, 100 MHz) δ (ppm): 175.30, 152.02, 134.97, 130.27, 129.94, 129.67, 129.64, 128.21, 123.30, 118.95, 115.19, 86.59, 85.49, 81.78, 71.00, 70.20, 56.06, 54.32, 52.07, 50.46, 49.45, 48.00, 45.52, 43.15, 39.81, 38.61, 38.02, 37.11, 34.77, 32.70, 32.25, 31.41, 28.66, 28.14, 28.01, 27.71, 26.36, 26.23, 25.09, 23.75, 18.25, 16.63, 16.45, 15.45. HR-MS (ESI) *m*/*z*: calcd. for C47H73N3O7S [M+H]^+^: 824.52420, found: 824.52173.

#### 3.3.8. (20S, 24S)-Epoxy-3β-*O*-[2-amino-(1-dansylamino)-5-dimethyl amino val-eryl]-dammarane-12β, 25-diol (**31**)

Yellow solid (yield 63.4%); m.p. 198.2~199.3 °C; [α${]}_{20}^{D}$ = 48.687 (c = 0.1, MeOH:CHCl_3_ = 3:1); ^1^H-NMR (CDCl_3_, 400 MHz) δ (ppm): 8.51 (d, *J* = 10.6 Hz, 1H, Ar-H), 8.29 (d, *J* = 8.7 Hz, 1H, Ar-H), 8.24 − 8.19 (m, 1H, Ar-H), 7.58 − 7.52 (m, 1H, Ar-H), 7.53 − 7.47 (m, 1H, Ar-H), 7.16 (d, *J* = 8.3 Hz, 1H, Ar-H), 5.75 (s, 1H, -OH), 4.44 (dd, *J* = 9.3, 7.2 Hz, 1H, -OCH-), 3.86 (dd, *J* = 10.8, 5.4 Hz, 1H, -OCH-), 3.51 (td, *J* = 10.3, 4.8 Hz, 1H, -OCH-), 3.23 (dd, *J* = 8.7, 4.1 Hz, 1H, -NH_2_CH-), 3.01 − 2.91 (m, 1H, -NCH_2_-), 2.87 (s, 6H, -CH3×2), 2.80 (ddd, *J* = 12.7, 7.6, 5.3 Hz, 1H, -NCH_2_-), 1.26 (s, 3H, -CH_3_), 1.23 (s, 3H, -CH_3_), 1.08 (s, 3H, -CH_3_), 0.99 (s, 3H, -CH_3_), 0.89 (s, 3H, -CH_3_), 0.88 (s, 3H, -CH_3_), 0.79 (s, 3H, -CH_3_), 0.75 (s, 3H, -CH_3_). ^13^C-NMR (CDCl_3_, 100 MHz) δ (ppm): 175.26, 152.04, 134.96, 130.28, 129.96, 129.68, 129.66, 128.22, 123.31, 118.95, 115.20, 87.46, 87.23, 81.81, 70.53, 70.13, 56.03, 54.33, 52.23, 50.19, 48.97, 48.86, 45.52, 43.15, 39.81, 38.04, 37.13, 34.72, 32.29, 32.02, 31.69, 31.3, 29.79, 28.96, 28.62, 28.13, 26.39, 25.13, 24.30, 23.78, 18.23, 17.85, 16.67, 16.41, 15.54. HR-MS (ESI) *m*/*z*: calcd. for C_47_H_73_N_3_O_7_S [M+H]^+^: 824.52420, found: 824.52191.

#### 3.3.9. (20S, 24R)-Epoxy-3β-*O*-[3-p-(N’-Fmoc) aromatic ring-2-alanine]-dammarane-12β, 25-diol (**34**)

White solid (yield 73.2%); m.p. 128.4~129.5 °C; [α${]}_{20}^{D}$ = 16.0 (c = 0.1, MeOH:CHCl3 = 3:1); ^1^H-NMR (CDCl_3_, 400 MHz) δ (ppm): 7.75 (d, *J* = 7.5 Hz, 2H, Ar-H), 7.59 (d, *J* = 7.4 Hz, 2H, Ar-H), 7.38 (t, *J* = 7.4 Hz, 2H, Ar-H), 7.29 (t, *J* = 7.4 Hz, 4H, Ar-H), 7.12 (d, *J* = 8.0 Hz, 2H, Ar-H), 6.99 (s, 1H, -NH-CO-), 4.49 (d, *J* = 6.4 Hz, 3H, -OCH-, -CH_2_-), 4.24 (t, *J* = 6.6 Hz, 1H, -OCH-), 3.83 (dd, *J* = 8.6, 6.6 Hz, 1H, -OCH-), 3.50 (td, *J* = 10.4, 4.4 Hz, 1H, -CH-), 2.97 (m, 2H, -CH_2_-), 1.26 (s, 3H, -CH_3_), 1.24 (s, 3H, -CH_3_), 1.08 (s, 3H, -CH_3_), 0.96 (s, 3H, -CH_3_), 0.87 (s, 3H, -CH_3_), 0.85 (s, 3H, -CH_3_), 0.80 (s, 6H, -CH3×2). ^13^C-NMR (CDCl_3_, 100 MHz) δ (ppm): 143.75, 141.32, 130.02, 127.75, 127.11, 124.97, 120.01, 86.49, 85.38, 82.11, 70.92, 70.16, 66.78, 56.02, 55.32, 51.99, 50.38, 49.35, 47.94, 47.11, 39.74, 38.56, 37.94, 37.02, 34.71, 32.59, 31.33, 31.18, 29.30, 28.57, 27.99, 27.92, 27.56, 26.10, 25.00, 23.57, 18.14, 16.44, 16.36, 15.37. HR-MS (ESI) *m*/*z*: calcd. for C_54_H_72_N_2_O_7_ [M+H]^+^: 861.5412, found: 861.53870.

#### 3.3.10. (20S, 24S)-Epoxy-3β-*O*-[3-p-(N’-Fmoc) aromatic ring-2-alanine]-dammarane-12β, 25-diol (**35**)

White solid (yield 73.2%); m.p. 135.4~135.6 °C; [α${]}_{20}^{D}$ = 6.0 (c = 0.1, MeOH:CHCl_3_ = 3:1); ^1^H-NMR (CDCl_3_, 400 MHz) δ (ppm): 7.76 (d, *J* = 7.5 Hz, 2H, Ar-H), 7.60 (d, *J* = 7.5 Hz, 2H, Ar-H), 7.39 (t, *J* = 7.4 Hz, 2H, Ar-H), 7.30 (td, *J* = 7.5, 1.1 Hz, 4H, Ar-H), 7.12 (d, *J* = 8.4 Hz, 2H, Ar-H), 6.81 (s, 1H, -NH-CO-), 5.74 (s, 1H, -OH), 4.55–4.45 (m, 3H, -NH-, -CH_2_-), 4.25 (t, *J* = 6.6 Hz, 1H, -OCH-), 3.86 (dd, *J* = 10.8, 5.4 Hz, 1H, -OCH-), 3.52 (td, *J* = 10.4, 4.8 Hz, 1H, -CH-), 2.92 (ddd, *J* = 81.4, 13.8, 6.4 Hz, 2H, -CH_2_-), 1.26 (s, 3H, -CH_3_), 1.22 (s, 3H, -CH_3_), 1.09 (s, 3H, -CH_3_), 1.00 (s, 3H, -CH3), 0.89 (s, 6H, -CH3×2), 0.83 (s, 3H, -CH_3_), 0.82 (s, 3H, -CH_3_). ^13^C-NMR (CDCl_3_, 100 MHz) δ (ppm): 174.55, 143.76, 141.36, 129.99, 127.77, 127.12, 124.95, 120.04, 87.15, 81.76, 70.48, 70.09, 66.80, 56.02, 55.71, 52.16, 50.15, 48.94, 48.83, 47.16, 40.12, 39.78, 38.56, 37.98, 37.09, 34.69, 32.21, 31.64, 29.70, 28.89, 28.54, 28.03, 25.10, 24.24, 23.64, 18.19, 17.77, 16.50, 16.35, 15.48. HR-MS (ESI) *m*/*z*: calcd. for C_54_H_72_N_2_O_7_ [M+H]^+^: 861.5412, found: 861.53802.

### 3.4. Antibacterial Activity

HA-MRSA 18–19, 18–20, and S. aureus ATCC 29213 were used to evaluate the antibacterial activity of all the compounds. HA-MRSA 18–19 and 18–20 were hospital-acquired MRSA strains (Chengdu, China). *S. aureus* ATCC 29213 was used as a control. Mueller-Hinton agar was prepared, with the final concentrations of the drug in each plate ranging from 128 to 0.008 μg/mL. Then, approximately 10^4^ colony-forming units (CFUs) of diluted bacterial solution were inoculated onto the prepared Mueller-Hinton agar. Then, HA-MRSA strains 18–15, 18–19, and 18–20 were used to evaluate selected compounds in a secondary assay.

### 3.5. Synergistic Antibacterial Activity

HA-MRSA 18–15 was used to evaluate the synergistic antibacterial activity of the selected compounds. First, the compounds were serially diluted from 32 to 1/32 times their MIC value and then a total of 100 μL of a mixed solution containing two compounds were added to the columns and rows of a 96-well plate. Then, 100 μL of a diluted bacterial solution containing approximately 5 × 10^4^ CFU were added to each well. The results were recorded after cultivation at 37 °C for 24 h. A 20 μL sample of the mixed solution from the 96-well plate was spread on drug-free Mueller-Hinton agar, and after cultivation at 37 °C for 24 h, the minimum bactericidal concentration (MBC) effect was determined.

## 4. Conclusions

Natural products are irreplaceable in the development of new drugs. Our previous research showed that ocotillol-type derivatives have good antibacterial activity against community-acquired MRSA (CA-MRSA). Recent research showed that ocotillol-type ginsenosides also have good antibacterial activity against HA-MRSA. In this research, ocotillol-type derivatives with nitrogen-containing groups were synthesized and their antibacterial activity was evaluated toward HA-MRSA in vitro. Compounds **20**–**23** and **28**–**31** exhibited potent antibacterial activity with MICs of 1–64 μg/mL. The results suggested that ocotillol-type compounds coupled with *N*-heterocycles containing a tertiary amine did not have improved antibacterial activity over ocotillol-type ginsenosides, and ocotillol-type derivatives with a connecting Fmoc were better than those with a connecting dansyl chloride. Compound **20** showed good antibacterial activity with an MIC of 4 μg/mL, and synergistic inhibitory effects when combined with conventional antibiotics CHL or KAN as shown by the FICI values of 0.5 and 0.3125, respectively. Compound **21** showed better antibacterial activity than compound **20** with an MIC of 1 μg/mL, and synergistic inhibitory effects when combined with CHL or KAN as shown by the FICI value of 0.375. The mechanism(s) responsible for the antibacterial and synergistic antibacterial effects of ocotillol-type derivatives against HA-MRSA strains has been inadequately studied. Our previous work showed that ocotillol-type derivatives were mainly distributed on the bacterial cell membrane; thus, the mechanism of the antibacterial potency and synergistic effect of ocotillol derivatives is probably related to the bacterial cell membrane. Compound **21** is a good probe that can be used to investigate the antibacterial and synergistic antibacterial mechanisms of ocotillol antibacterial derivatives.

## Figures and Tables

**Table 1:** In vitro antibacterial activity of ocotillol-type derivatives (MIC: μg/mL).

Cpd.	Antimicrobial Screening MIC (μg/mL)		
	MRSA 18–19	MRSA 18–20	*S. aureus* ATCC 29213
**8**	>128	>128	>128
**9**	>128	>128	>128
**10**	>128	>128	>128
**11**	>128	>128	>128
**12**	>128	>128	>128
**13**	>128	>128	>128
**14**	>128	>128	>128
**15**	>128	>128	>128
**20**	4	4	16
**21**	1	1	1
**22**	8	2	4
**23**	4	2	8
**28**	64	32	32
**29**	32	64	32
**30**	4	8	32
**31**	4	8	8
**34**	>128	>128	>128
**35**	>128	>128	>128
**4**	>128	>128	>128
**5**	64	64	64
**Levofloxacin**	8	0.125	0.25

MIC: minimal inhibit concentration.

**Table 2:** Synergistic effect of CHL with compounds **20** and **21** against MRSA 18–15.

	MIC (μg/mL)	MBC (μg/mL)	FICI
	MRSA 18–15	MRSA 18–15	MRSA 18–15
CHL	8	>32	-
**20**	4	>64	-
**21**	2	>128	-
**20** + CHL	2	NE	0.5
**21** + CHL	1	NE	0.375

CHL: chloramphenicol; NE: no effect; MIC: minimal inhibit concentration; MBC: minimum bactericidal concentration.; FICI: Fractional inhibitory concentration index.

**Table 3:** Synergistic effect of KAN with compounds **20** and **21** against MRSA 18–15.

	MIC (μg/mL)	MBC (μg/mL)	FICI
	MRSA 18–15	MRSA 18–15	MRSA 18–15
**KAN**	2	>8	-
**20**	4	>64	-
**21**	1	>128	-
**20 + KAN**	0.5	NE	0.3125
**21 + KAN**	0.25	NE	0.375

CHL: chloramphenicol; NE: no effect; MIC: minimal inhibit concentration; MBC: minimum bactericidal concentration.; FICI: Fractional inhibitory concentration index.

## Data Availability

Data are contained within the article and the supplementary material.

## Supplementary Materials

Supplementary materials are available online.

## Sample Availability

Samples of the compounds are available from the authors.
