# Supplementary material for: Design, Synthesis, and Antibacterial Evaluation of Novel Ocotillol Derivatives and Their Synergistic Effects with Conventional Antibiotics
Source: Molecules. 2021 Oct 1;26(19):5969. doi: 10.3390/molecules26195969 (PMC8512374; doi:10.3390/molecules26195969)
Supplement: Supplementary file 1 [file molecules-26-05969-s001.zip › molecules-1389900-supplementary.pdf]

Supplementary Materials

Design, Synthesis and Antibacterial Evaluation of Novel Ocotillol Derivatives and Their Synergistic Effects with Conventional Antibiotics

Doudou Zhang[1,#], Yucheng Cao[1,#], Kaiyi Wang[1,#], Zhuoyue Shi[1], Ruodong Wang[1], Qingguo Meng[1,*] and Yi Bi[1,*]

School of Pharmacy, Key Laboratory of Molecular Pharmacology and Drug Evaluation (Yantai University), Ministry of Education, Collaborative Innovation Center of Advanced Drug Delivery System and Biotech Drugs in Universities of Shandong, Yantai University, Yantai 264005 (P.R. China)
koikoi1998@yeah.net (D.-D.Z.); 404777634@qq.com (Y.-C.C.); 836633510@qq.com (K.-Y.W.); 1161324642@qq.com (Z.-Y.S.); 1297694491@qq.com (R.-D.W.)
*Correspondence: qinggmeng@163.com (Q.-G.M.); beeyee_413@163.com (Y.B.); Tel.: +86-0535-6706022 (Q.-G.M.); +86-0535-6706285 (Y.B.)
[#] These authors have equally contributed to the work.

Table of Contents

Copies of ^{1}H NMR, ^{13}C NMR and HR-MS spectra ....Pages S3-S20

Copies of ^{1}H NMR, ^{13}C NMR and HR-MS spectra

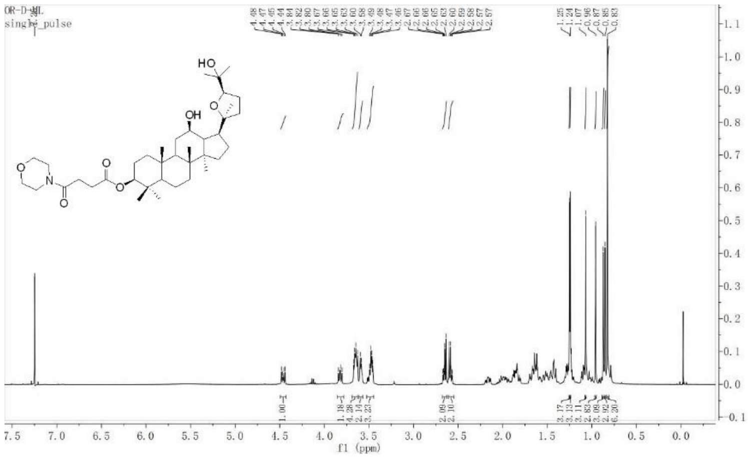

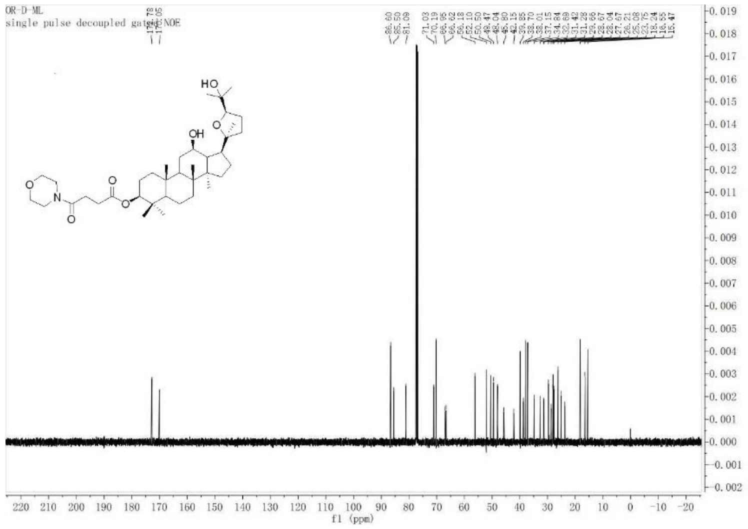

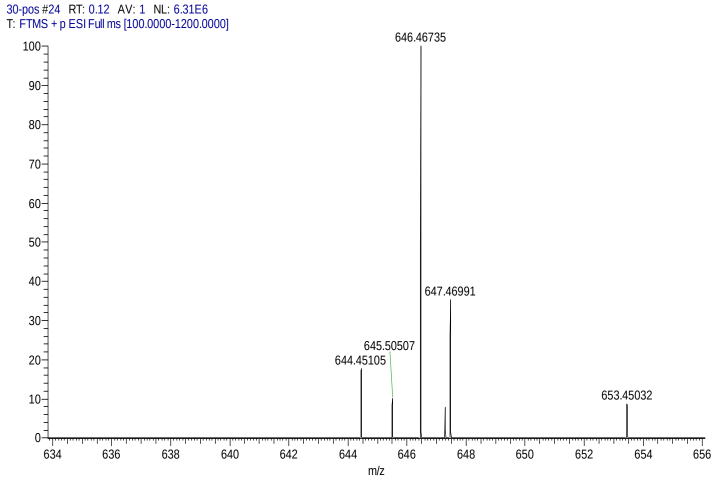

^{1}H NMR, ^{13}C NMR and HR-MS (ESI) spectra of compound **8**

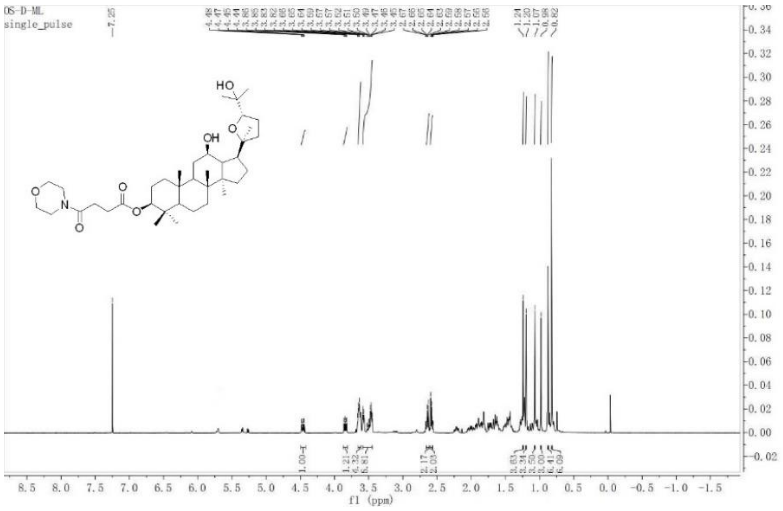

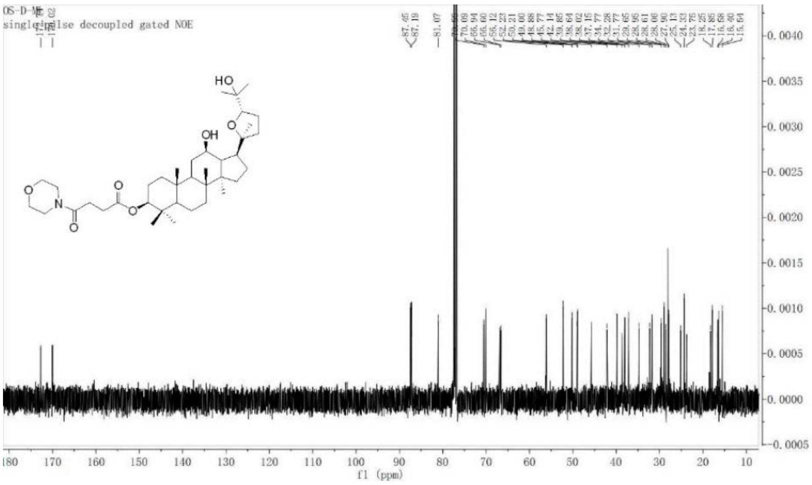

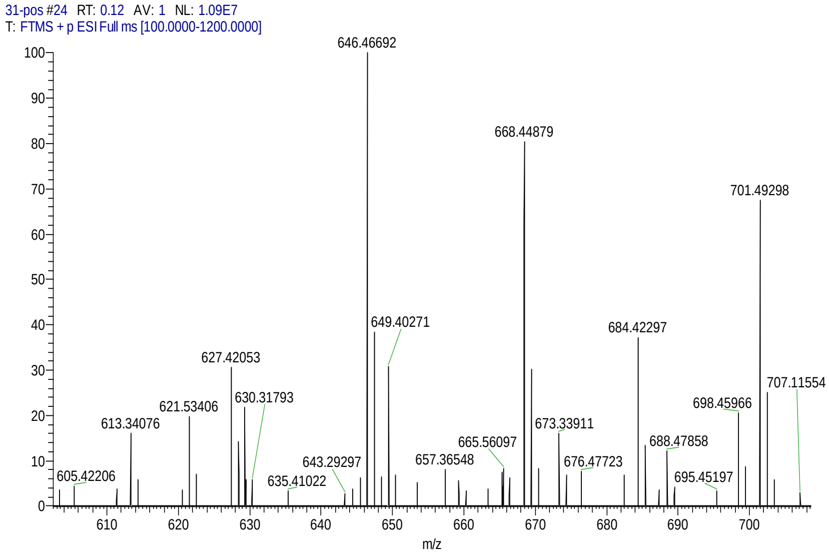

^{1}H NMR, ^{13}C NMR and HR-MS (ESI) spectra of compound **9**

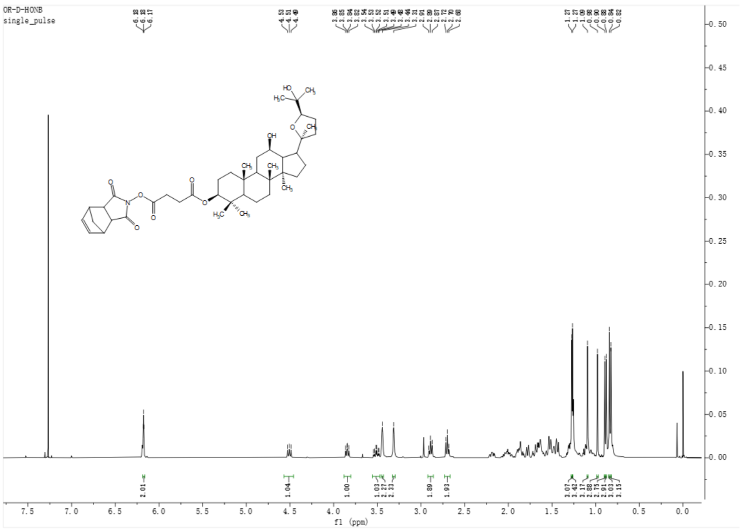

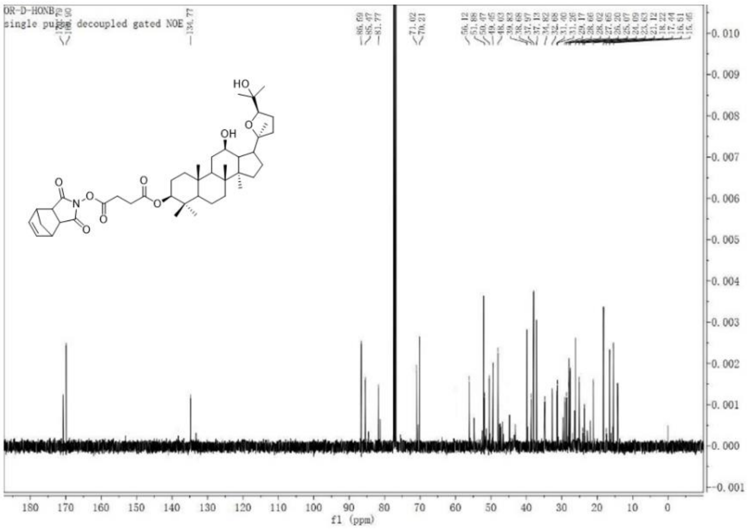

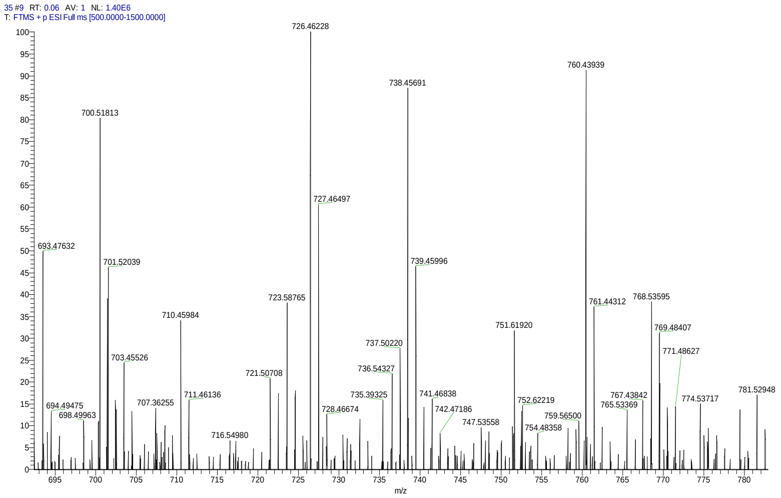

^{1}H NMR, ^{13}C NMR and HR-MS (ESI) spectra of compound **10**

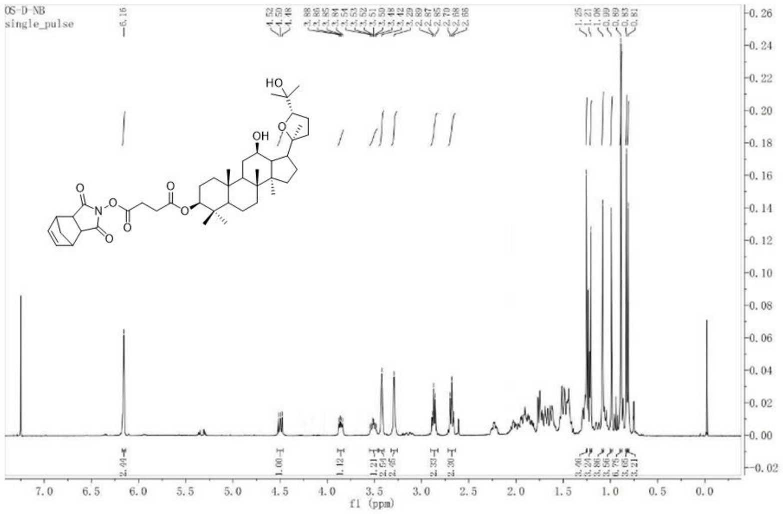

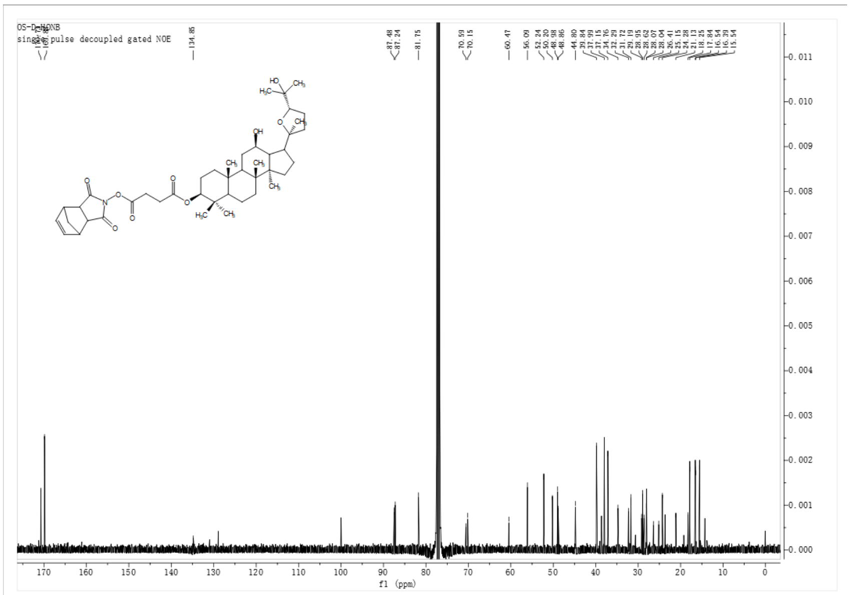

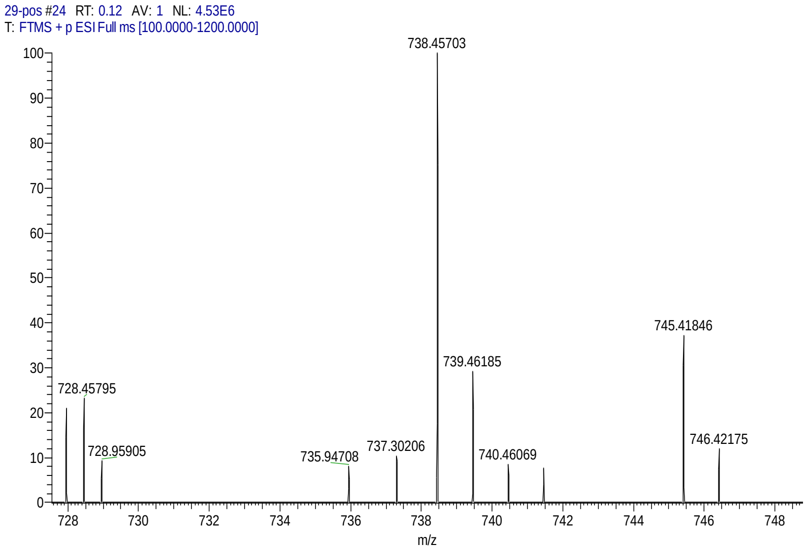

^{1}H NMR, ^{13}C NMR and HR-MS (ESI) spectra of compound **11**

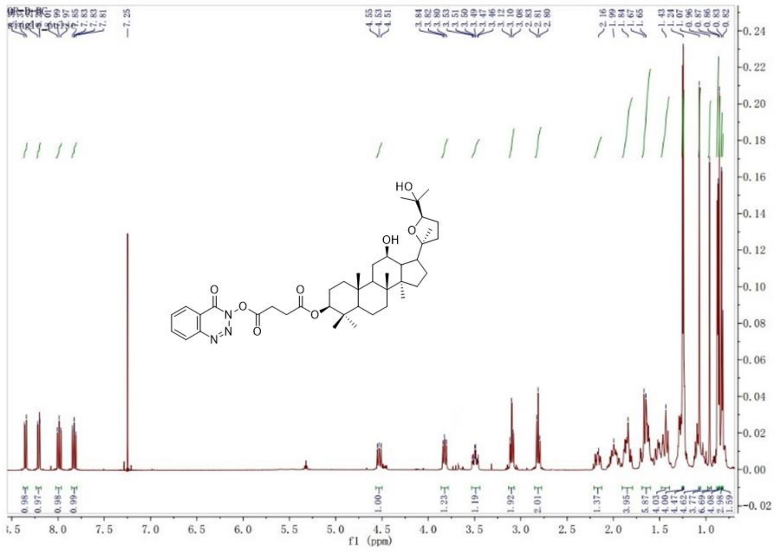

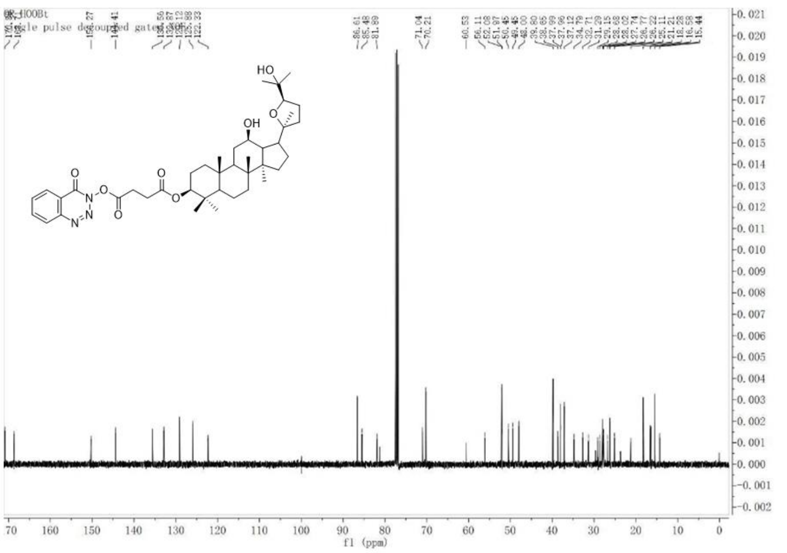

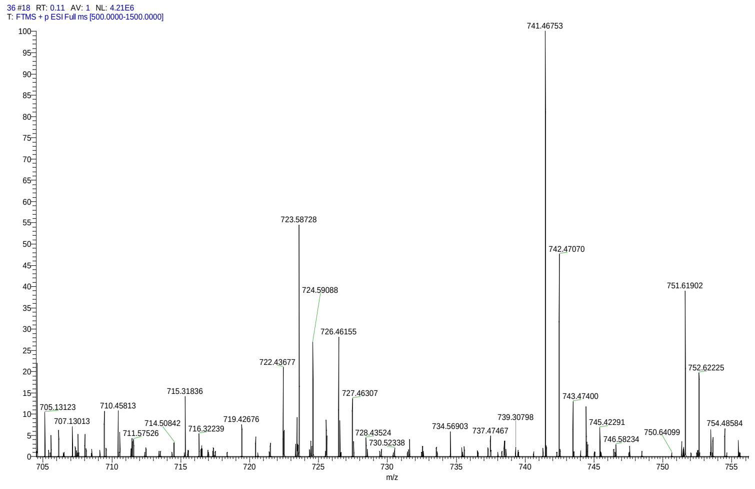

^{1}H NMR, ^{13}C NMR and HR-MS (ESI) spectra of compound **12**

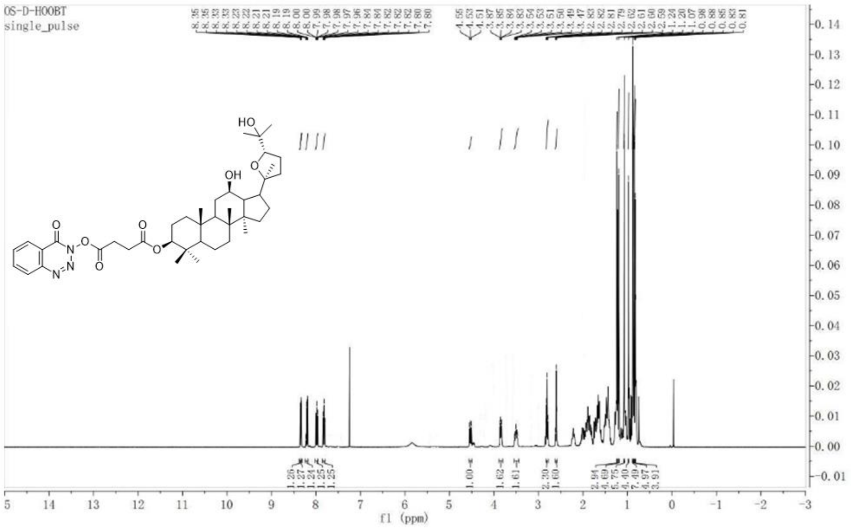

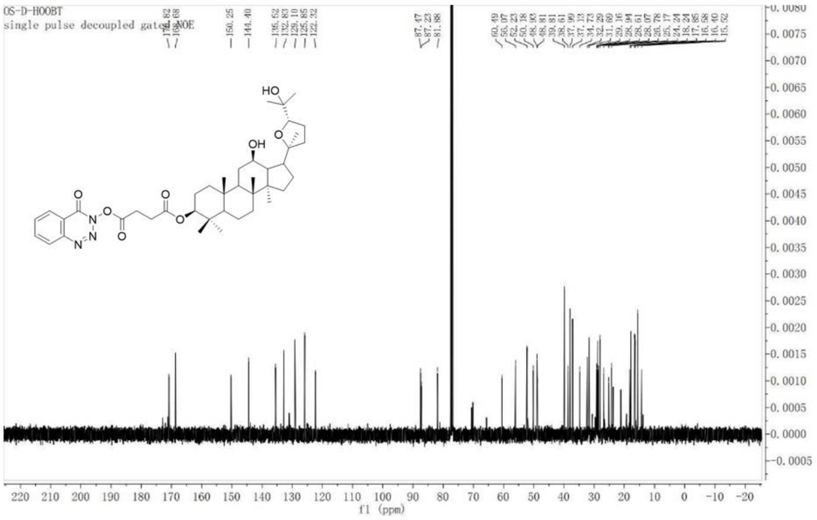

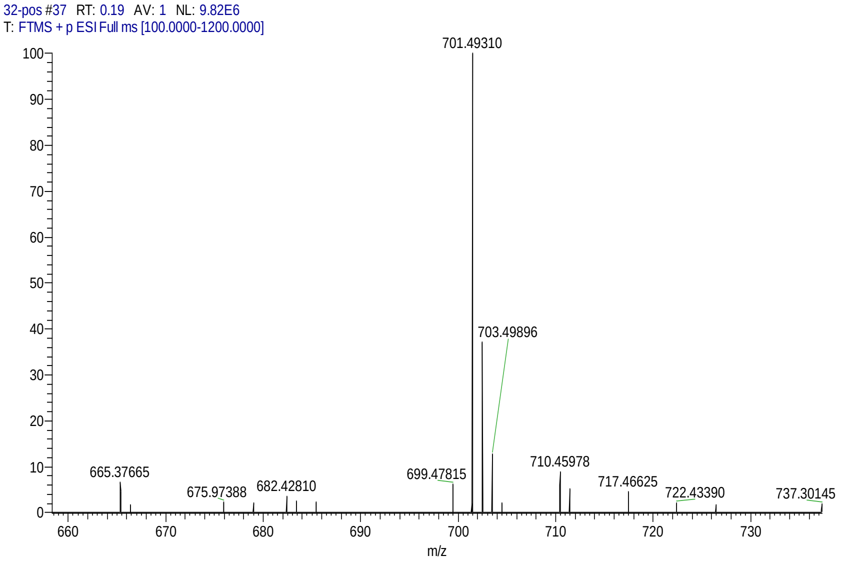

^{1}H NMR, ^{13}C NMR and HR-MS (ESI) spectra of compound **13**

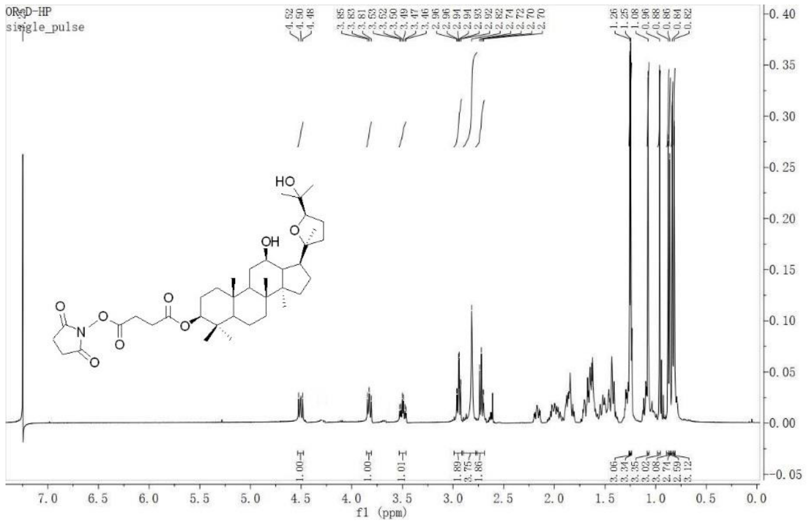

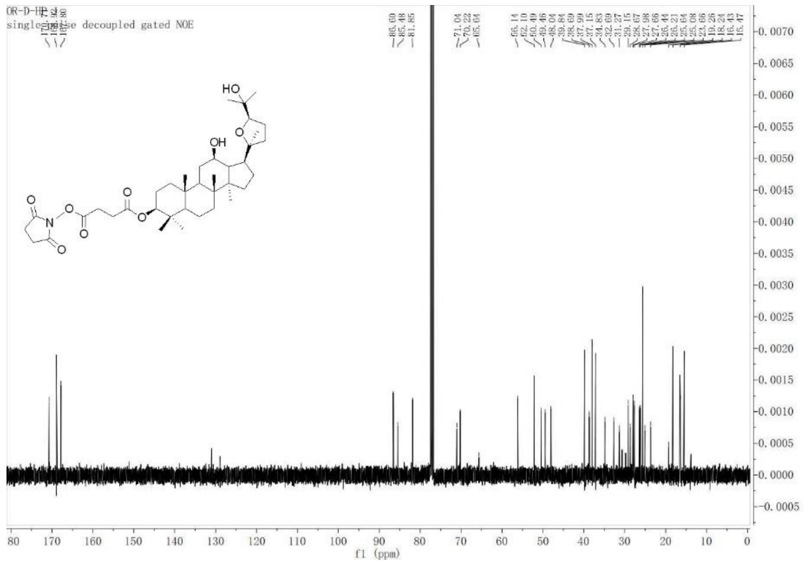

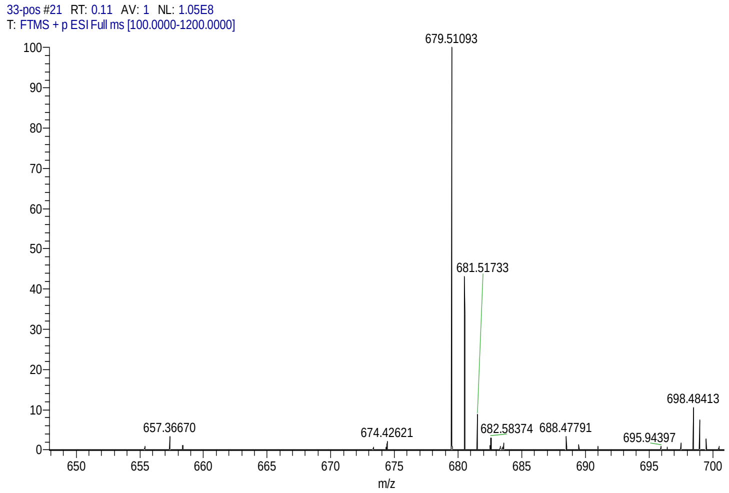

^{1}H NMR, ^{13}C NMR and HR-MS (ESI) spectra of compound **14**

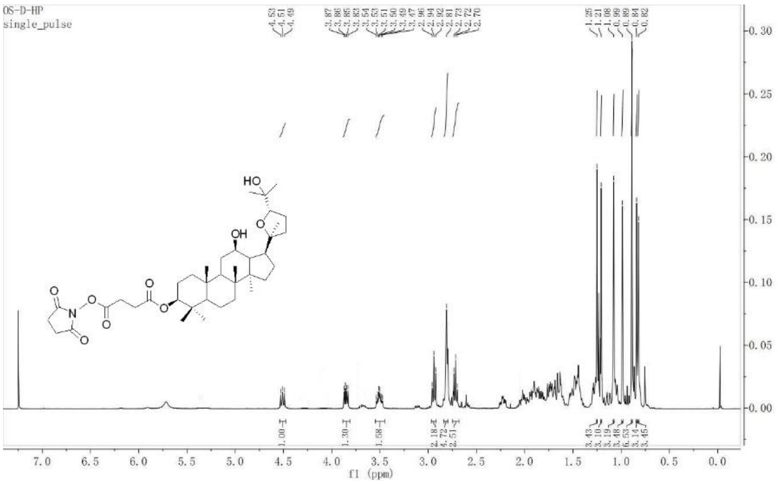

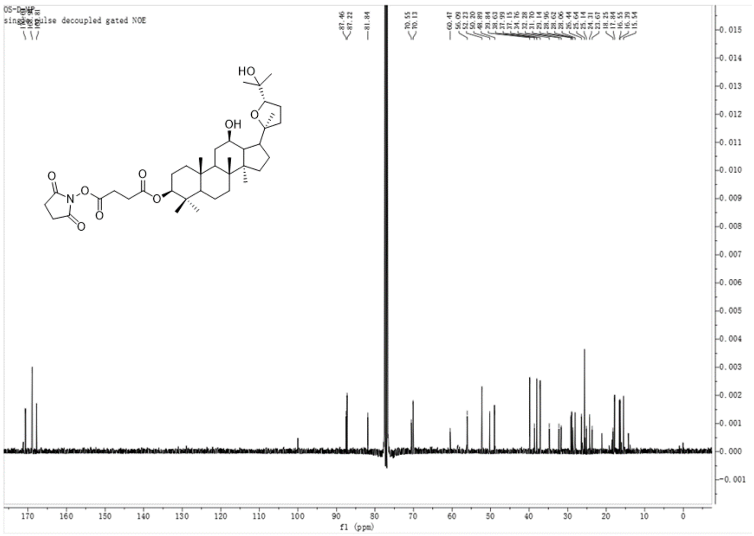

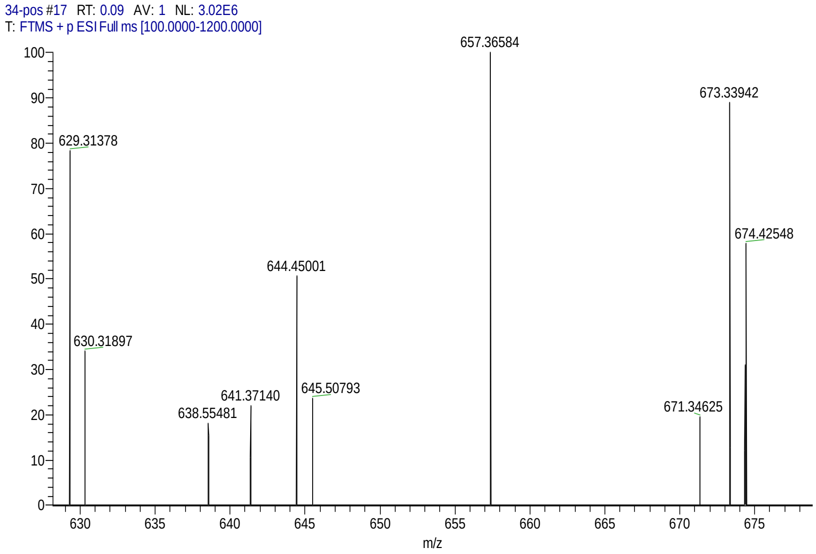

^{1}H NMR, ^{13}C NMR and HR-MS (ESI) spectra of compound **15**

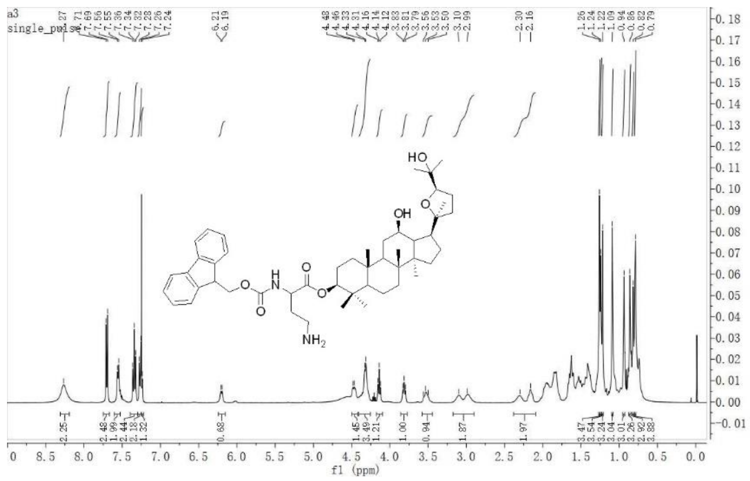

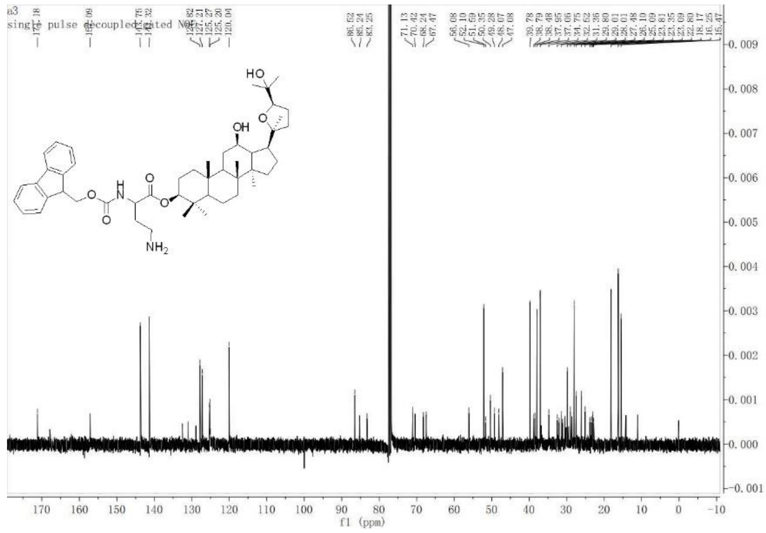

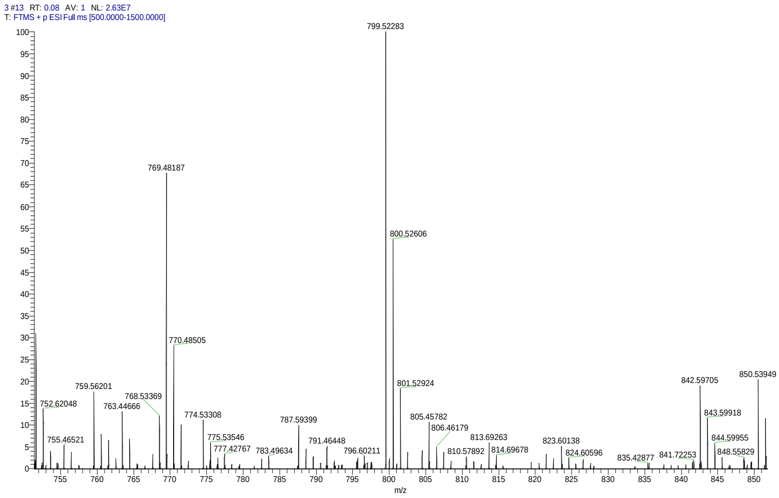

^{1}H NMR, ^{13}C NMR and HR-MS (ESI) spectra of compound **20**

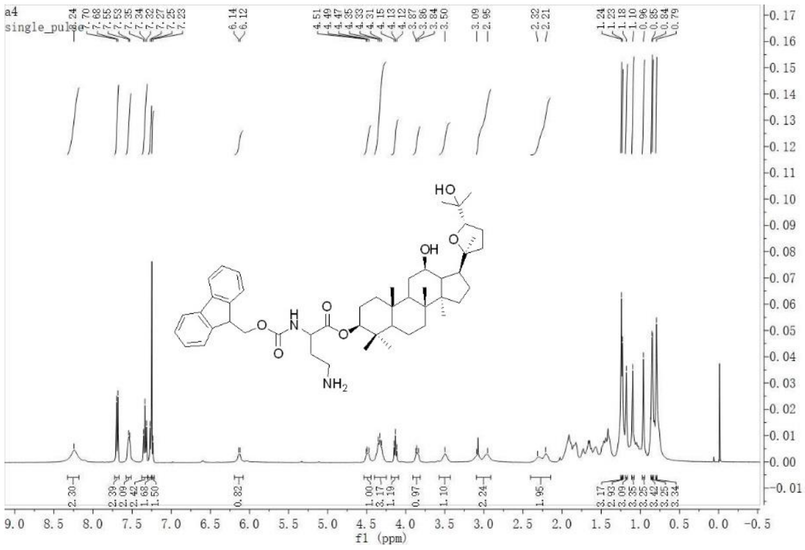

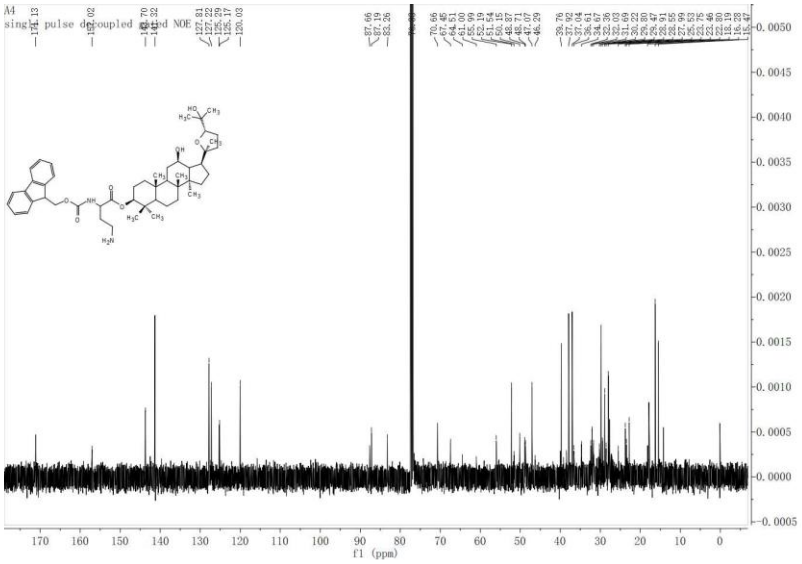

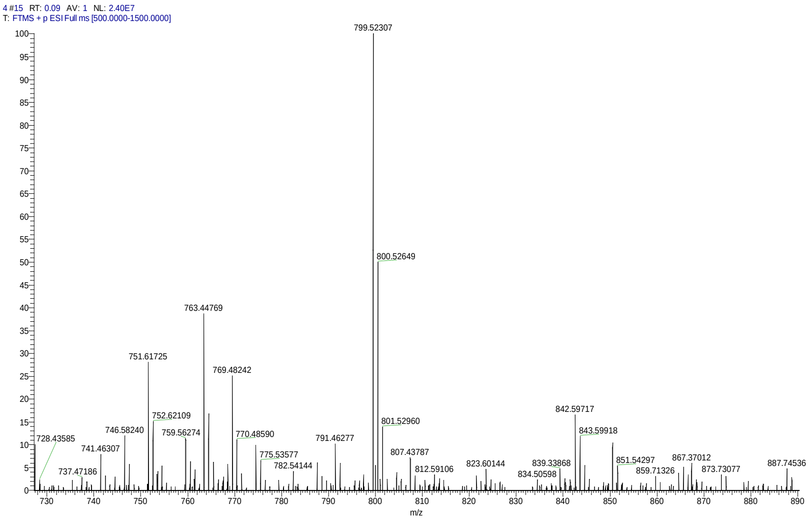

^{1}H NMR, ^{13}C NMR and HR-MS (ESI) spectra of compound **21**

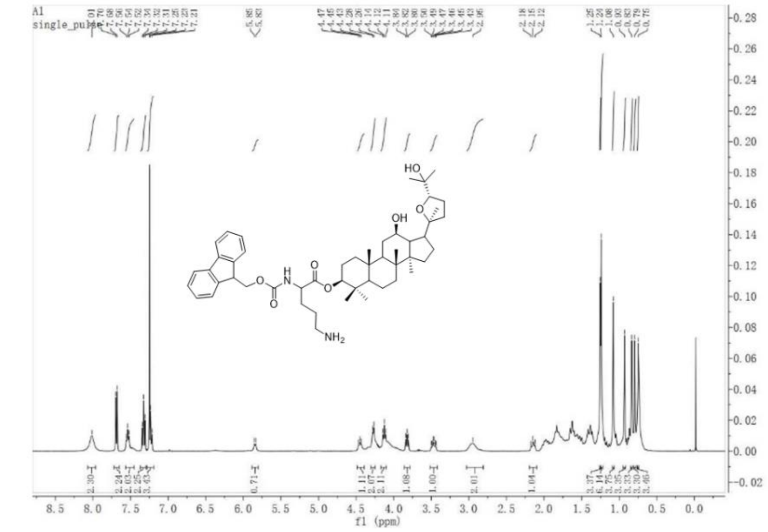

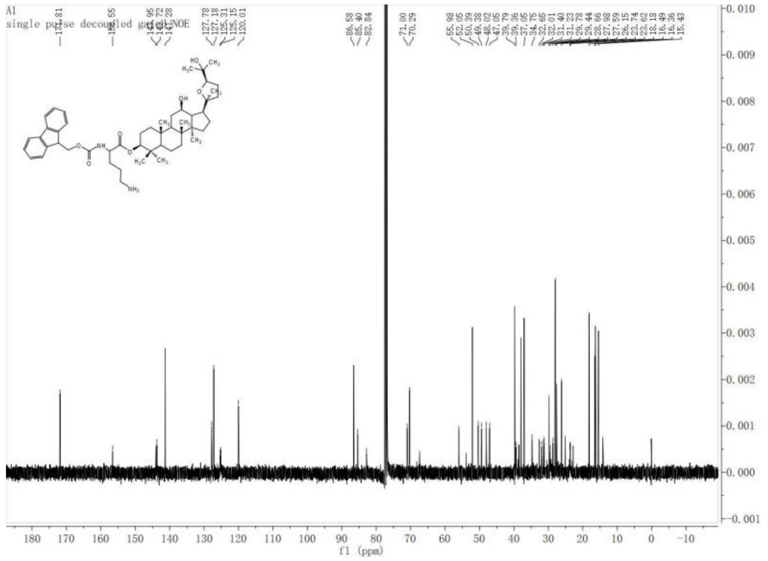

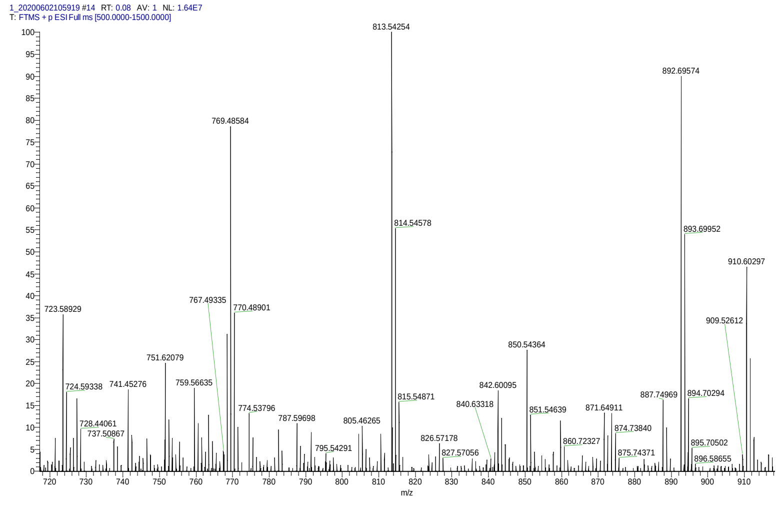

^{1}H NMR, ^{13}C NMR and HR-MS (ESI) spectra of compound **22**

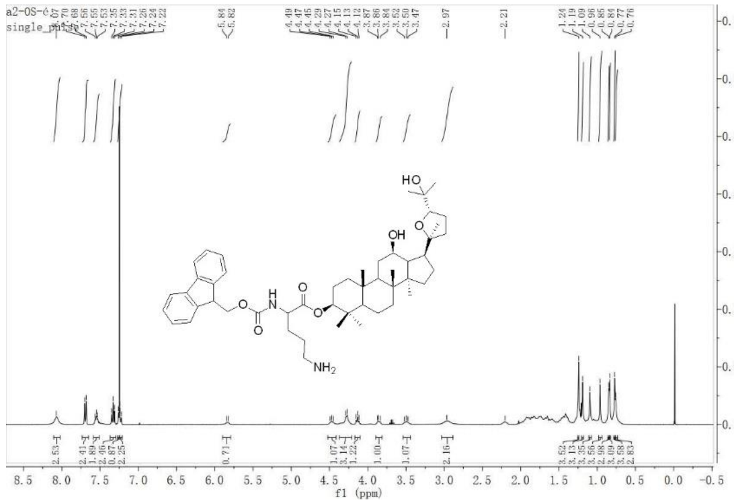

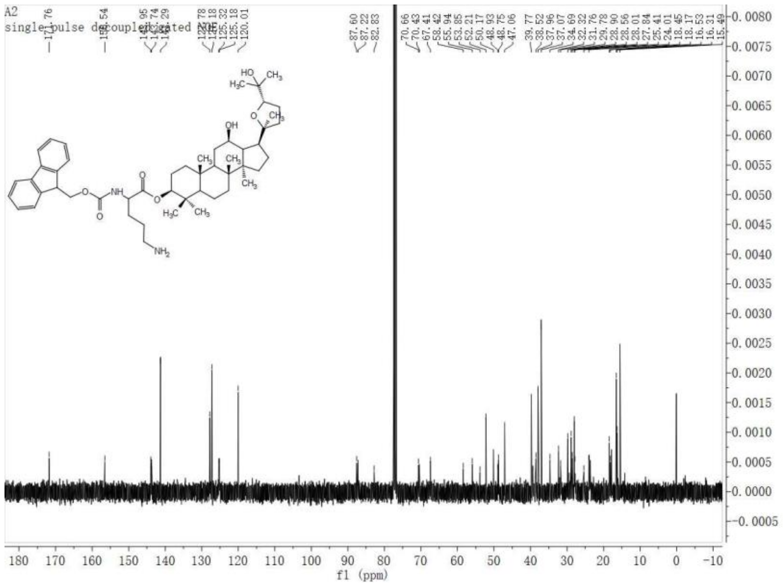

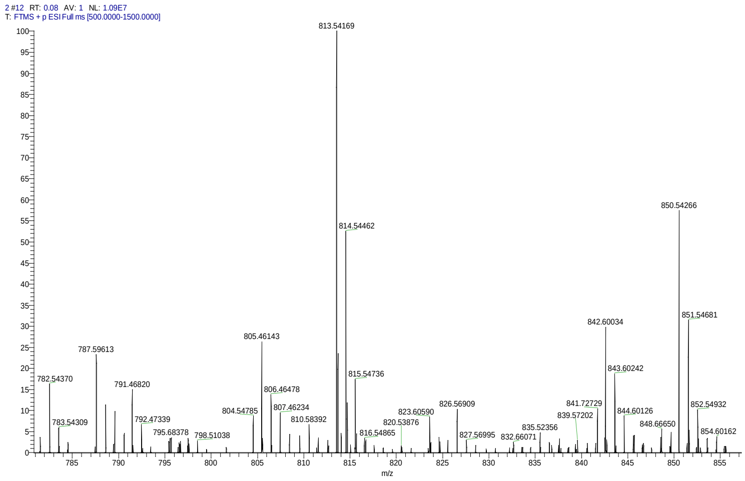

^{1}H NMR, ^{13}C NMR and HR-MS (ESI) spectra of compound **23**

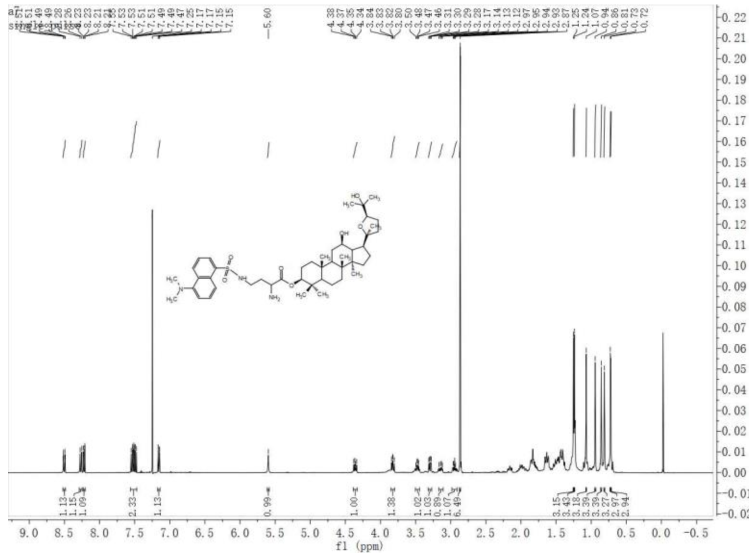

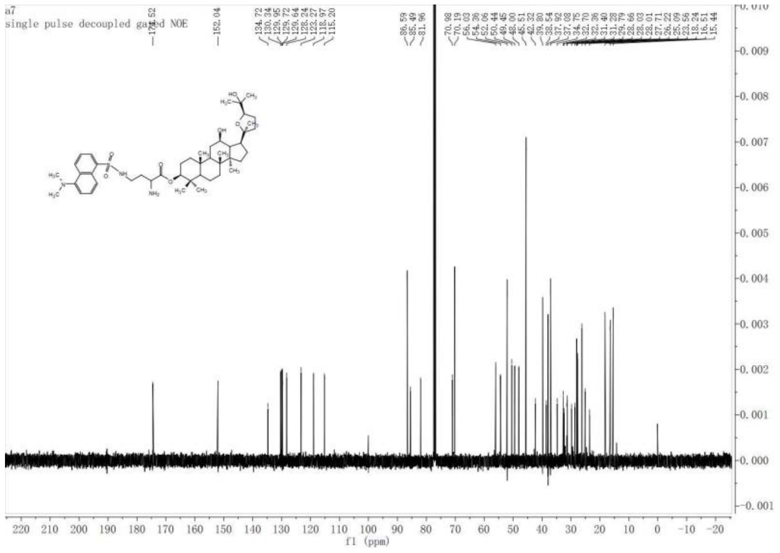

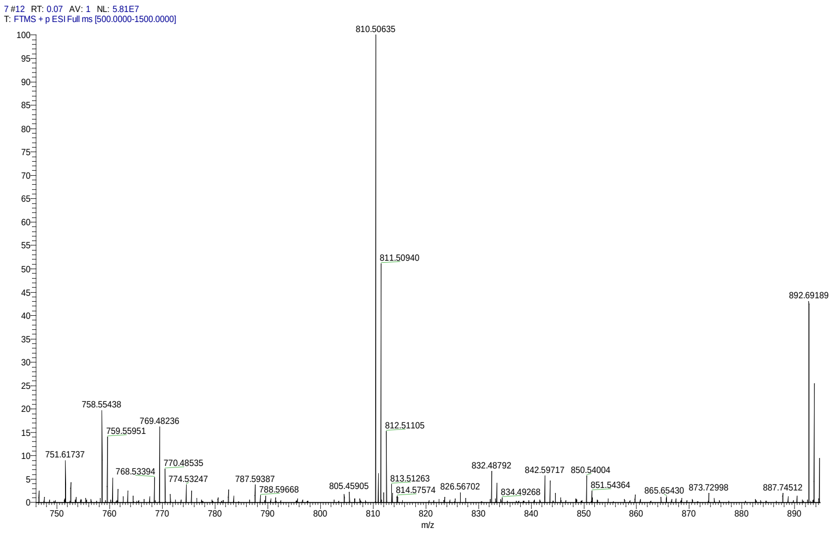

^{1}H NMR, ^{13}C NMR and HR-MS (ESI) spectra of compound **28**

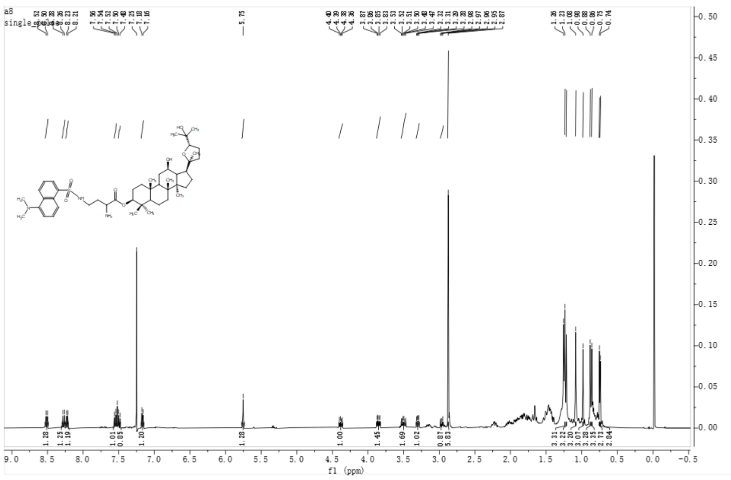

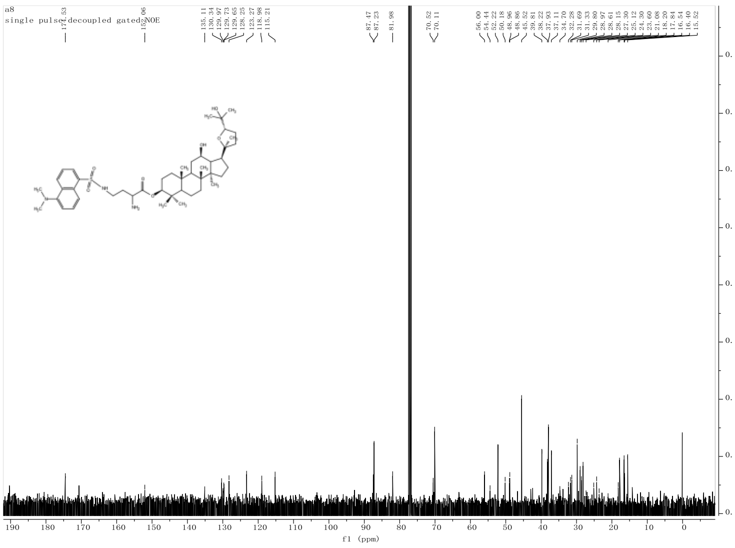

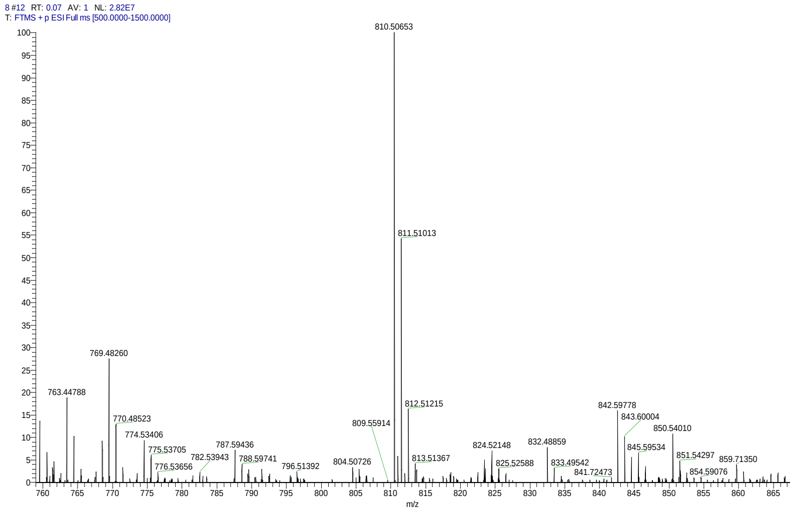

^{1}H NMR, ^{13}C NMR and HR-MS (ESI) spectra of compound **29**

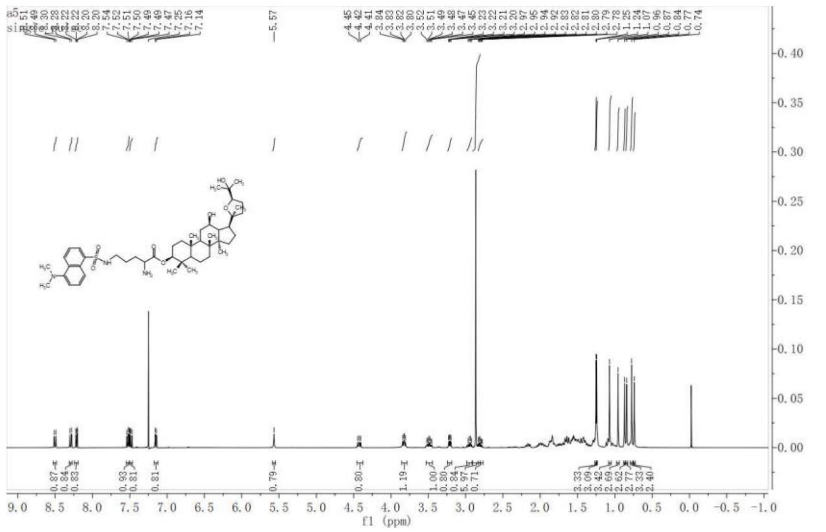

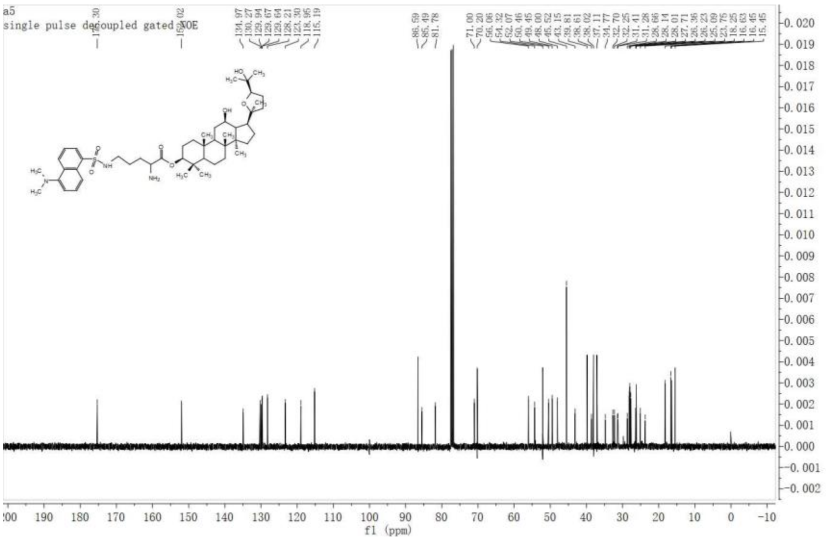

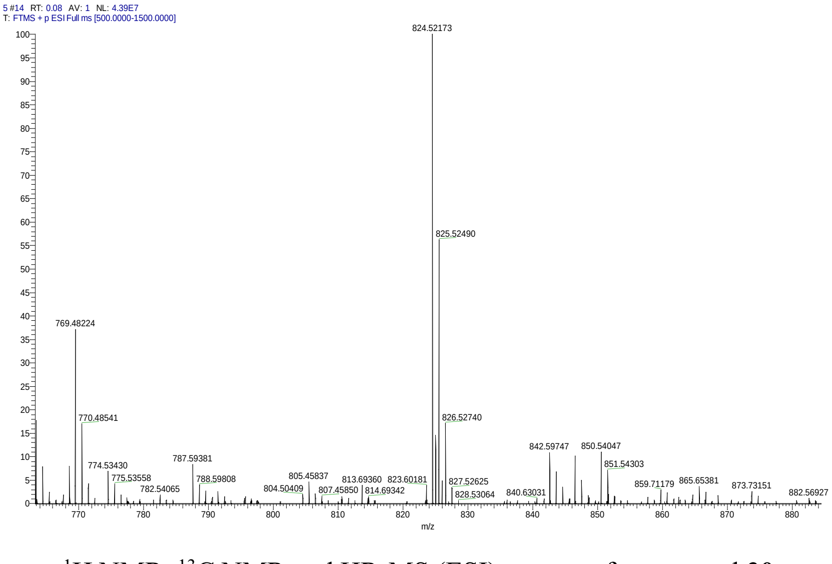

^{1}H NMR, ^{13}C NMR and HR-MS (ESI) spectra of compound **30**

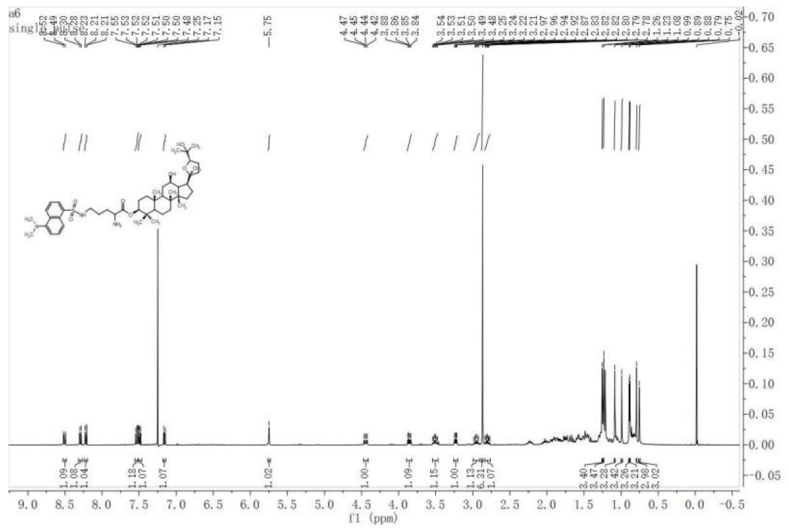

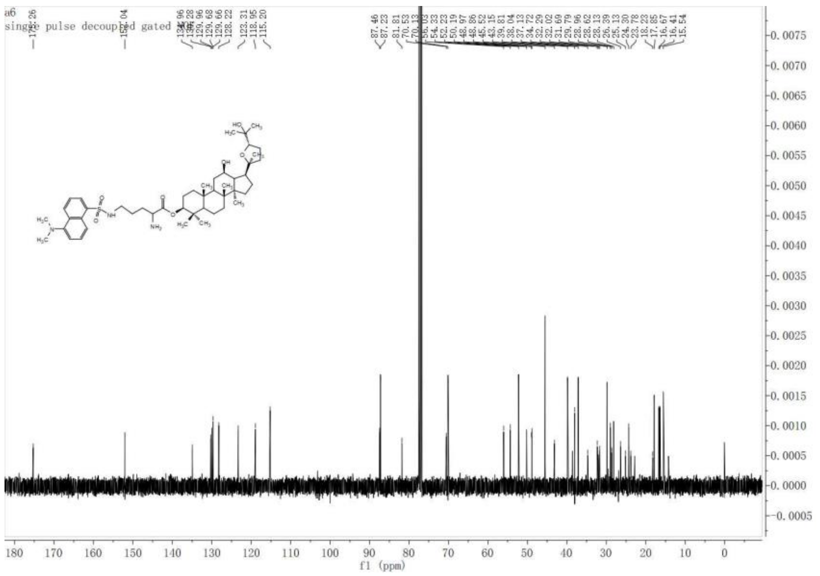

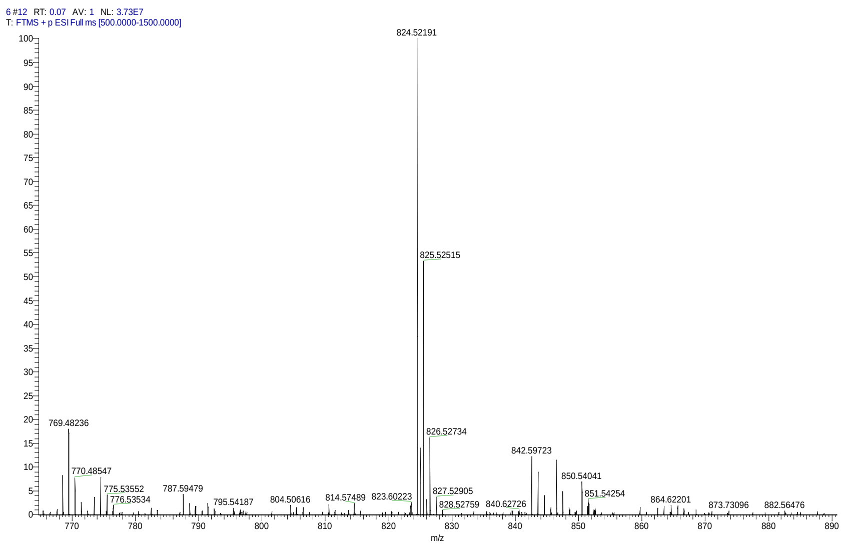

^{1}H NMR, ^{13}C NMR and HR-MS (ESI) spectra of compound **31**

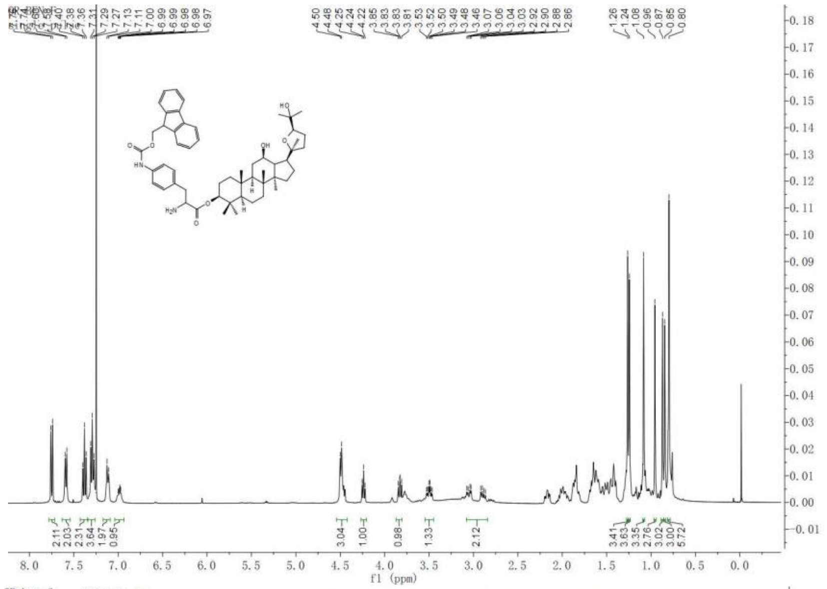

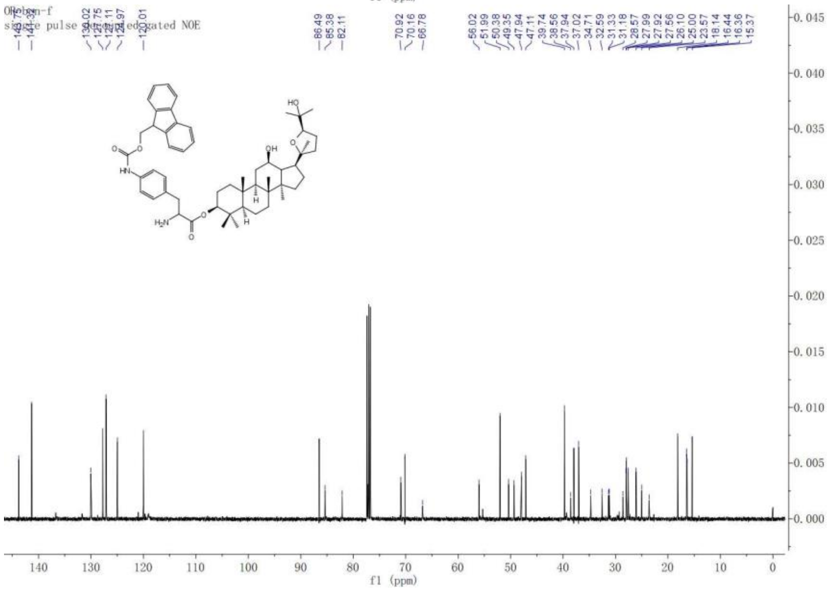

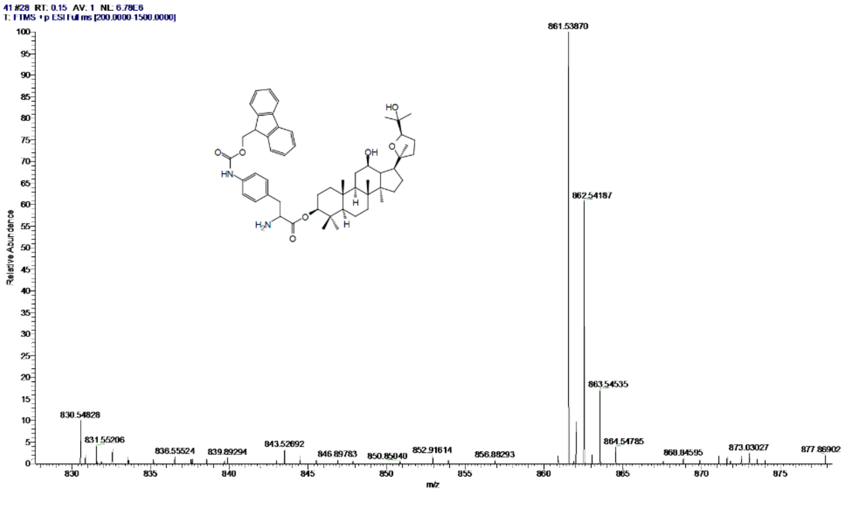

^{1}H NMR, ^{13}C NMR and HR-MS (ESI) spectra of compound **34**

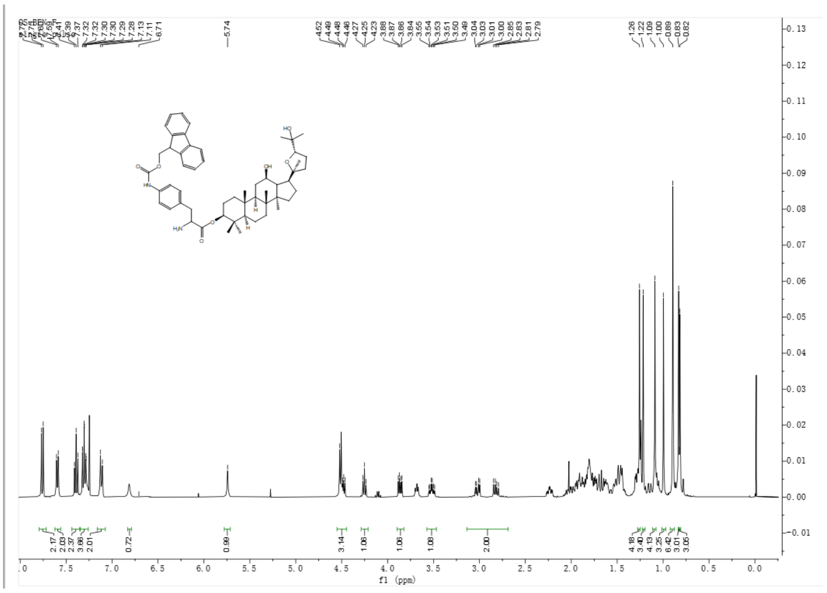

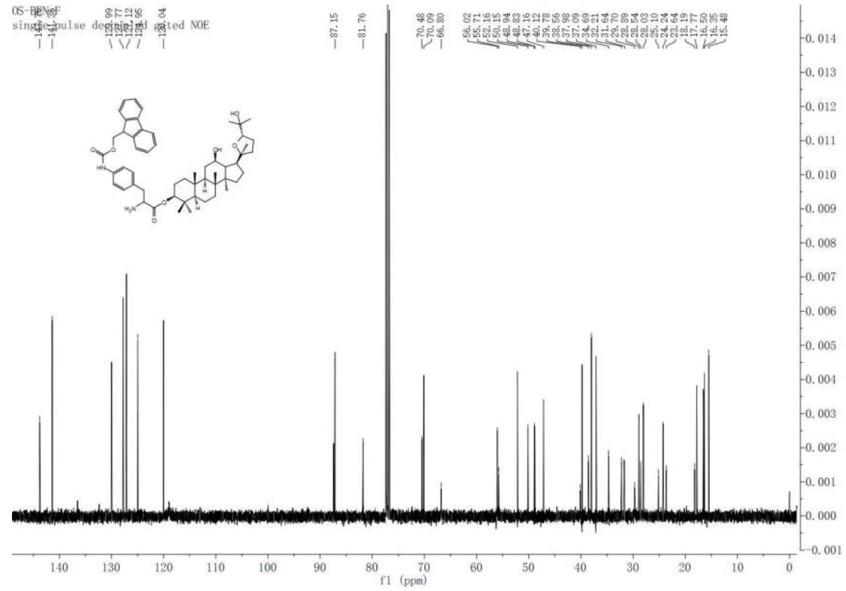

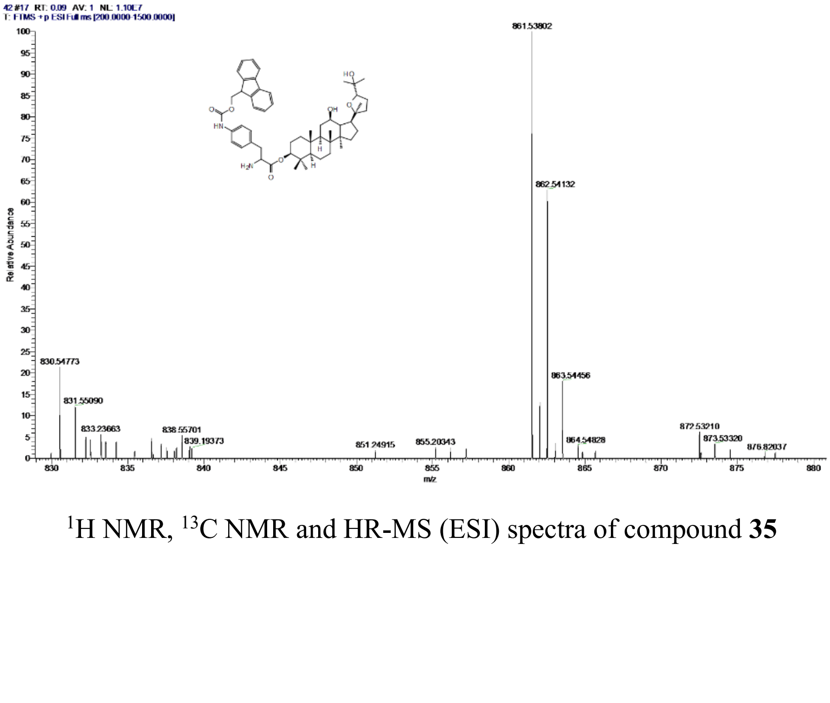

^{1}H NMR, ^{13}C NMR and HR-MS (ESI) spectra of compound **35**
